# Diseases of Iberian ibex (*Capra pyrenaica*)

**DOI:** 10.1007/s10344-023-01684-0

**Published:** 2023-06-01

**Authors:** Marta Valldeperes, Paloma Prieto Yerro, Jorge Ramón López-Olvera, Paulino Fandos, Santiago Lavín, Ramón C. Soriguer Escofet, Gregorio Mentaberre, Francisco Javier Cano-Manuel León, José Espinosa, Arián Ráez-Bravo, Jesús M. Pérez, Stefania Tampach, Josep Estruch, Roser Velarde, José Enrique Granados

**Affiliations:** 1 Servei d’Ecopatologia de Fauna Salvatge (SEFaS), Universitat Autònoma de Barcelona, 08193 Barcelona, Bellaterra Spain; 2 Wildlife Ecology & Health Group (WE&H), Barcelona, Spain; 3 Parque Natural de las Sierras de Cazorla, Segura y Las Villas, C/ Martínez Falero 11, 23470 Cazorla Jaén, Spain; 4 Grupo de Investigación RNM 118. Biología de Especies Cinegéticas y Plagas, Jaén, Spain; 5 C/Ocaña 44 2° D, 28047 Madrid, Spain; 6 Estación Biológica de Doñana (CSIC), Av. Américo Vespucio sn, 41092 Sevilla, Spain; 7 Departament de Ciència Animal, Universitat de Lleida, 25198 Lleida, Spain; 8 Junta de Andalucía, Departamento de Actuaciones en el Medio Natural, Av. Joaquina Eguaras 2, 18013 Granada, Spain; 9 Departamento de Sanidad Animal, Facultad de Veterinaria and Instituto de Ganadería de Montaña (CSIC-ULE), Universidad de León, 24071 León, Spain; 10 Department of Animal and Plant Biology, and Ecology, Jaén University, Campus Las Lagunillas, 23071 Jaén, Spain; 11 Parque Nacional y Parque Natural de Sierra Nevada. Ctra., Antigua de Sierra Nevada Km 7, Pinos Genil, 18191 Granada, Spain

**Keywords:** Disease, Epidemiology, Iberian ibex, Management, Pathogens, Sarcoptic mange

## Abstract

Iberian ibex (*Capra* *pyrenaica*) is an ecologically and economically relevant medium-sized emblematic mountain ungulate. Diseases participate in the population dynamics of the species as a regulating agent, but can also threaten the conservation and viability of vulnerable population units. Moreover, Iberian ibex can also be a carrier or even a reservoir of pathogens shared with domestic animals and/or humans, being therefore a concern for livestock and public health. The objective of this review is to compile the currently available knowledge on (1) diseases of Iberian ibex, presented according to their relevance on the health and demography of free-ranging populations; (2) diseases subjected to heath surveillance plans; (3) other diseases reported in the species; and (4) diseases with particular relevance in captive Iberian ibex populations. The systematic review of all the information on diseases affecting the species unveils unpublished reports, scientific communications in meetings, and scientific articles, allowing the first comprehensive compilation of Iberian ibex diseases. This review identifies the gaps in knowledge regarding pathogenesis, immune response, diagnostic methods, treatment, and management of diseases in Iberian ibex, providing a base for future research. Moreover, this challenges wildlife and livestock disease and wildlife population managers to assess the priorities and policies currently implemented in Iberian ibex health surveillance and monitoring and disease management.

## Introduction

Disease can be defined as damage or deterioration affecting normal functions, including responses to extrinsic and intrinsic factors such as nutrition, climate, toxic agents, micro- and macroparasites, and congenital defects, alone or combined (Wobeser 1994). Wildlife can share diseases with humans, known as zoonoses, as well as with domestic animals. Thus, wildlife diseases are relevant for human and domestic livestock health, since wildlife can act as a disease reservoir, but they are also a matter of concern for wildlife conservation, particularly in endangered species or populations (Gortázar et al. 2007, 2016; Cunningham et al. 2017).

Iberian ibex (*Capra pyrenaica*) is a medium-sized mountain ungulate, formerly endemic to the Iberian Peninsula but recently introduced in the northern Pyrenees in France (Manceau et al. 1999; Granados et al. 2001; Pérez et al. 2002; Acevedo and Cassinello 2009; Alados and Escós 2017; Garnier 2021). Although two of the four subspecies originally described are extinct, most of the Iberian ibex evolutionary significant units and populations are currently expanding and spreading or at least stable (Granados et al. 2001; Pérez et al. 2002; Acevedo and Cassinello 2009; Alados and Escós 2017). However, some ibex populations are vulnerable to risk factors that threaten their viability and conservation, including habitat alteration and fragmentation, inadequate management, overabundance, low genetic diversity, competition with domestic and wild ungulates, human disturbance, and diseases (Manceau et al. 1999; Pérez et al. 2002; Acevedo and Cassinello 2009). On the other hand, the expansion of this species increases the risk of direct or indirect transmission of pathogens with other wild and domestic animals and/or humans (Gortázar et al. 2007, 2016; Lawson et al. 2021).

Research and reviews on wildlife diseases, conservation, and management are required (Illarietti et al. 2023). The objective of this review is to compile the currently available knowledge on diseases of Iberian ibex. The diseases are presented in order of relevance, dealing first with those with proven disease, epizootic, and/or mortality effects on free-ranging Iberian ibex populations (“Infectious diseases causing disease and mortality in Iberian ibex populations” section) and then the diseases included in the Spanish national wildlife health surveillance program (Lawson et al. 2021; Gobierno de España et al. 2022; “Diseases under targeted surveillance in the Iberian ibex” section), followed by other diseases investigated and/or reported in Iberian ibex but with little or unknown effects in population health and demography (“Other diseases” section). Finally, the diseases favored by captivity are specifically mentioned, since captive Iberian ibex populations are subjected to particular conditions and health risks (Granados et al. 1996b; Espinosa et al. 2017c).

## Infectious diseases causing symptomatology and mortality with demographic impact in Iberian ibex populations

### Sarcoptic mange

Sarcoptic mange is considered an emerging or re-emerging zoonotic disease, caused by the skin-burrowing mite *Sarcoptes scabiei*. This mite is considered a single species with host taxon-specific varieties, which are morphologically undistinguishable although they have high host specificity and low capacity of cross-infection (Fain 1968, 1978; Pence et al. 1975; Arlian et al. 1984b; Walton et al. 1999; Zahler et al. 1999; Pence and Ueckermann 2002; Rasero et al. 2010). Additionally, within each taxon, geographic differences can also be found (Berrilli et al. 2002).

Despite such specificity, interspecific transmission of *S. scabiei* has been reported within the same taxon among domestic and wild species both for ungulates and carnivores (Kutzer 1966; Samuel 1981; Ibrahim and Abu-Samra 1987; León-Vizcaíno et al. 1992; Lavín et al. 2000a; Menzano et al. 2007; Valldeperes et al. 2021). Cross-transmission related to predation and scavenging with the establishment of specific predator/scavenger-prey cycles has also been documented (Gakuya et al. 2011; Oleaga et al. 2011; Matsuyama et al. 2019). Being a zoonotic disease, cross-transmission between humans and different animal species also occurs, either directly or mediated through domestic animals, especially pets (Arlian et al. 1984b; Morsy et al. 1994; Mitra et al. 1995; Menzano et al. 2004; Walton and Currie 2007; Rentería Solís et al. 2014; Pisano et al. 2019; Moroni et al. 2022b). Nevertheless, such interspecific transmissions among hosts from different taxa are usually self-limited both in the individual host and in the population and thus do not lead to outbreaks or demographic consequences. On the other hand, although they usually act as dead-end hosts, these temporal host species could be relevant as parasite reservoirs for the original natural host species (Menzano et al. 2004; Rossi et al. 2007).

Sarcoptic mange is an emerging panzootic in wildlife, reported in up to 148 domestic and wild animal mammal species, including humans, causing wildlife population declines and livestock economic losses (Pence and Ueckermann 2002; Walton et al. 2004; Escobar et al. 2022). Among the wild host species, the effect of sarcoptic mange has been dramatic when affecting mountain caprine populations in Eurasia, being considered a health emergency at the wild/domestic caprine interface in Europe (Rossi et al. 2019; Pérez et al. 2021). Particularly, sarcoptic mange has been described in most of the Iberian ibex populations, frequently associated with dramatic demographic declines (Fandos 1991; León-Vizcaíno et al. 1999; Valldeperes et al. 2018a, b).

#### Etiology

*Sarcoptes scabiei* has a monoxenous life cycle, developing all the stages in a single host. Once fertilized, females burrow galleries in the host skin and lay up to 50 eggs during their 4–6 weeks lifetime, at a rhythm of 3–4 eggs per day (Walton and Currie 2007; Arlian and Morgan 2017). After three to eight days, hexapodial larvae exit the eggs and burrow into short galleries known as pouches. There they molt consecutively to protonymphs in 3 to 4 days, to tritonymphs in 2 to 3 days, and finally into adults after 2 or 3 days more (Arlian and Vyszenski-Moher 1988; Bornstein et al. 2001). Apparently, less than 1% of the eggs complete this cycle to become adults (Mellanby 1944).

Survival of *S. scabiei* off the host is limited because the mites cannot maintain their water balance. Mite survival increases with lower temperatures and higher humidity, although rising temperatures up to 35° enhance mite mobility (Mellanby et al. 1942; Arlian et al. 1989). Congelation at − 25 °C and 50% of relative humidity for 90 min killed 100% of *S. scabiei* var. *canis* mites (Arlian et al. 1984a). The lifespan of adult females and nymphs doubles that of male mites (Arlian et al. 1989).

#### Symptomatology, pathogenesis, and immune response

**Fig. 1:**
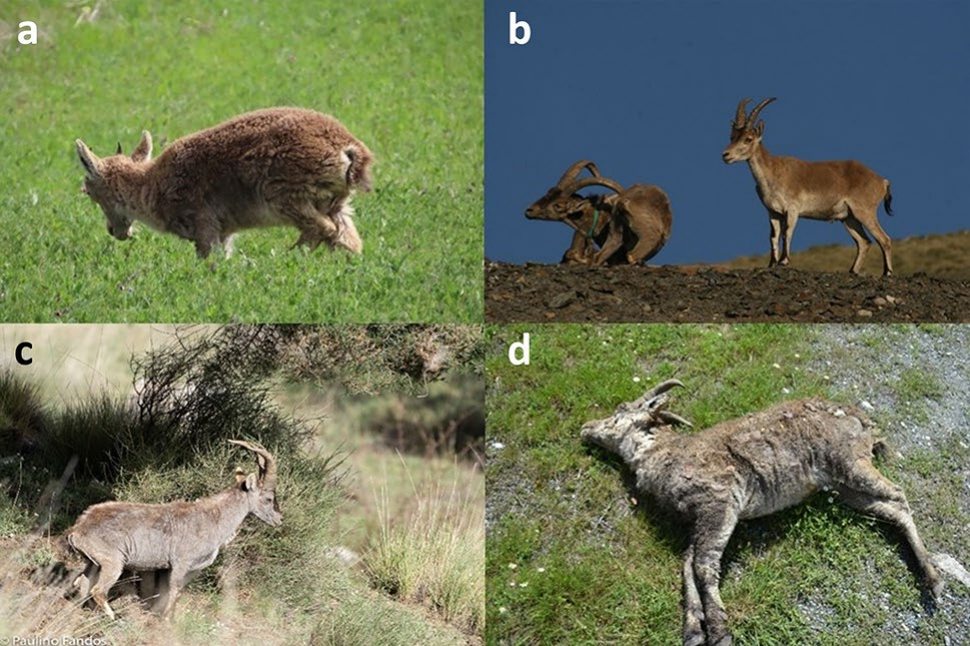
Iberian ibexes affected by sarcoptic mange with skin lesions of different severity. **a** Female ibex with mild sarcoptic mange (grade I). **b** Scratching mangy adult male collared for monitoring (left) accompanied by a healthy young male. **c** Young male ibex with severe sarcoptic mange (grade III–IV). **d** Young male ibex died because of sarcoptic mange with 100% of the body surface affected (grade IV)

Clinical signs vary depending on the host species and the individual immune status (Pence and Ueckermann 2002), but sarcoptic mange consistently induces skin local inflammatory response directly related to the severity of the disease and the number of mites (Petersen et al. 2004). Clinical signs include pruritus, seborrhoea, erythema, papules, and alopecia, progressing to hyperkeratosis, skin thickening, and crusts when the disease becomes chronic, followed by secondary bacterial infections (Bornstein et al. 2001; Nakagawa et al. 2009; Niedringhaus et al. 2019; Swe et al. 2014; Espinosa et al. 2017d). These secondary bacterial infections are favored by the immunosuppressing action of the mite and the scratching lesions (Mika et al. 2012; Swe et al. 2014), originating deep recurring pyodermas that can evolve to septicaemia (McCarthy et al. 2004; Nakagawa et al. 2009). In Iberian ibex, five categories regarding the extension of body surface affected by lesions compatible with sarcoptic mange have been defined, namely, 0, without apparent lesions; grade I, skin surface affected ≤ 25%; grade II, skin surface affected between 25 and 50%; grade III, skin surface affected between 50 and 75%; and grade IV, skin surface affected > 75% (Pérez et al. 2011a; Ráez-Bravo et al. 2015) (Fig. 1). However, *S. scabiei* infestation has further multisystemic consequences beyond the local skin lesions (Espinosa et al. 2020), including a reduction in body condition, hematological and serum biochemical disorders (Perez et al. 2015), increase in oxidative stress with a decrease in the antioxidant status (Saleh et al. 2011; Espinosa et al. 2017b; Naesborg-Nielsen et al. 2022), and/or inflammatory acute phase protein response (Ráez-Bravo et al. 2015; Pastor et al. 2019), with amyloid deposits in parenchymal organs (Espinosa et al. 2017d, 2020). All these changes contribute to the pathogenesis of the disease, reducing survival and/or hampering the recovery of ibexes in chronic stages of sarcoptic mange. The intense pruritus and continuous scratching related to the massive release of histamine and other antigenic compounds impair feeding and resting, causing constant stress and energetic imbalance (Bates 1997). This negative energy balance is further worsened by heat loss due to general hair loss (Cross et al. 2016; Simpson et al. 2016; Süld et al. 2017) and feeding impairment by severe lesions in the lips making food apprehension difficult (Abu-Samra et al. 1981; León-Vizcaíno et al. 1999), leading overall to weight loss independently of the availability of resources, which is more severe in males (Carvalho et al. 2015; López-Olvera et al. 2015). The progressive decline of physical condition also affects reproduction (Davies 1995; Sarasa et al. 2011; Espinosa et al. 2017a), skeletal development, and body and horn growth (Serrano et al. 2007; Pérez et al. 2011b).

As the pathogenic effects of the mites, host immune response against sarcoptic mange is also both local and systemic. Systemically, *S. scabiei* infestation induces antibody production, namely, immunoglobulins E and G, although this systemic immune humoral response is correlated with the extension of the lesions, acting as an indicator of inflammation rather than being protective (Lastras et al. 2000; Rodríguez-Cadenas et al. 2010; Pérez et al. 2015; Ráez-Bravo et al. 2016; Naesborg-Nielsen et al. 2022). Similarly, the acute-phase protein response is also correlated with the severity of sarcoptic mange in Iberian ibex (Ráez-Bravo et al. 2015). Although the effects of sarcoptic mange in male Iberian ibex are more severe (López-Olvera et al. 2015), the systemic humoral immune response is more intense in females, either naïve or in reinfestations (Sarasa et al. 2010). Thus, although previous exposition to the mite may induce a certain resistance to reinfection, with a faster and more intense systemic immune response (Van Neste 1986; Arlian et al. 1994a, b; Arlian et al. 1996, 1995; Taringan 2014), in Iberian ibex, this seems to be true for males rather than for females (Sarasa et al. 2010). The systemic cellular immune response consists of an increase in leukocyte count, including an increase in T lymphocytes, neutrophils, and eosinophils in the blood (Pérez et al. 2015).

In the skin, sarcoptic mange induces a local immune cellular response with macrophages and T lymphocytes and, to a lesser extent, B lymphocytes and plasma cells, overall resembling a type I and/or type IV hypersensitivity immune response (Lalli et al. 2004; Salvadori et al. 2016; Bhat et al. 2017; Martínez et al. 2020; Naesborg-Nielsen et al. 2022). The predominant cellular type and the immune response pathway and genes activated by the mite seem to determine the clinical outcome of scabietic humans and animals. Consequently, in wildlife species, the local immune cellular response can determine the probability of individual survival and the population demographic impact. Overall, the individuals developing a less intense but more efficient local skin inflammatory cellular response have more probability of developing mild clinical mange, preventing the progression to severer disease (Bhat et al. 2017; Ráez-Bravo 2019). Immunosuppressant effects by the mite through the downregulation of the immune response pathways and genes to facilitate the colonization of the skin have been suggested (Lalli et al. 2004; Mika et al. 2012; Ráez-Bravo 2019). The trade-off between the immunosuppressing action of the mite and the local and systemic immune response of the hosts determines the distribution and abundance of mites in the affected skin, which is not homogeneous (Castro et al. 2016; Arlian and Morgan 2017); the number of mites carried by a host; the proportion of skin surface affected; the severity of the disease; and, consequently, the clinical outcome (Pérez et al. 2011a; Ráez-Bravo 2019).

#### Diagnosis

There is not yet a diagnostic method for sarcoptic mange with the ideal sensitivity and specificity (Chandler and Fuller 2019; Pérez et al. 2021). While visual diagnosis is the most basic, extended, and long-term used method for sarcoptic mange monitoring on the field and has therefore become the reference (Pérez et al. 2011a) (Fig. 1), both false negatives and false positives occur (Valldeperes et al. 2019; Pérez et al. 2021). Such limitations led to the recommendation of completing visual diagnosis on the field with the detection of mites and/or eggs in skin scrapings or biopsies digested with potassium hydroxide (Pérez et al. 2011a; Valldeperes et al. 2019), unveiling an 87% sensitivity and a 61% specificity for visual diagnosis on the field (Valldeperes et al. 2019). However, this method requires capturing and handling the ibex or retrieving the carcass for sampling, and while 100% specific, it can also produce false negatives (Rambozzi et al. 2004; Walton and Currie 2007; Walter et al. 2011).

Further advances in sarcoptic mange diagnosis have been proposed to overcome limitations and improve sensitivity, specificity, and/or applicability on the field, including (1) molecular techniques to detect genetic material of *S. scabiei* through polymerase chain reaction (PCR) (Mounsey et al. 2012; Alasaad et al. 2015), which allows phylogenetic analyses (Ueda et al. 2019; Moroni et al. 2021); (2) detection of the immunoglobulin G generated by the host species after contact with *S. scabiei* (Puigdemont et al. 2002; Rambozzi et al. 2004; Bornstein et al. 2006; Casais et al. 2007; Oleaga et al. 2008; Millán et al. 2012; Haas et al. 2015a; Ráez-Bravo et al. 2016), which allows retrospective epidemiologic studies (Haas et al. 2018); (3) camera-trapping, useful for detecting mange in naïve or suspected areas reducing surveillance effort but unreliable to assess prevalence and with the same probability of false negatives and positives as visual diagnosis (Oleaga et al. 2011; Haas et al. 2015b; Brewster et al. 2017; Carricondo Sánchez et al. 2017; Saito and Sonoda 2017); (4) thermal imaging, based in the increased body heat radiation of mangy individuals due to hair loss and skin inflammation, with low sensitivity beyond a distance of 100 m and therefore mostly useful only with handled individuals (Cross et al. 2016; Arenas et al. 2002; Granados et al. 2011), where other reliable specific and sensitive alternatives exist; and (5) trained dogs, which have successfully detected dead chamois or severely affected by sarcoptic mange in the Alps, apparently with high sensitivity and 100% specificity (Alasaad et al. 2012). However, none of these diagnostic methodologies have been either validated or standardized yet.

Finally, the non-specific increases of acute-phase proteins and oxidative stress induced by sarcoptic mange have been proposed to diagnose and monitor sarcoptic mange in wildlife (Rahman et al. 2010; Ráez-Bravo et al. 2015; Espinosa et al. 2017b). However, these indicators can also increase for other disorders (Pastor et al. 2019) and therefore cannot be considered diagnostic for an individual, although they can be helpful to monitor wildlife populations where sarcoptic mange is the only or the main disease circulating.

#### Epidemiology

Direct contact between infested and naïve hosts is the main transmission route of *S. scabiei*. The individuals with chronic lesions and higher percentages of affected skin carrying more mites are the main source of infestation for their conspecifics (Arlian and Vyszensky-Moher 1988; Pérez et al. 2011a). Nevertheless, indirect transmission through fomites also exists. Sarcoptic mange epidemiology is further determined by both intrinsic factors, such as host sex, age, body condition, and social behavior (González-Candela and León-Vizcaíno 1999; Rahbari et al. 2009; Carvalho et al. 2015; López-Olvera et al. 2015), and extrinsic factors, such as temperature, relative humidity, and seasonality (Pérez et al. 1997a; Rahbari et al. 2009; Pérez et al. 2011a, 2017; Iacopelli et al. 2020; Martín-Monedero et al. 2022; Pérez et al. 2022).

**Fig. 2:**
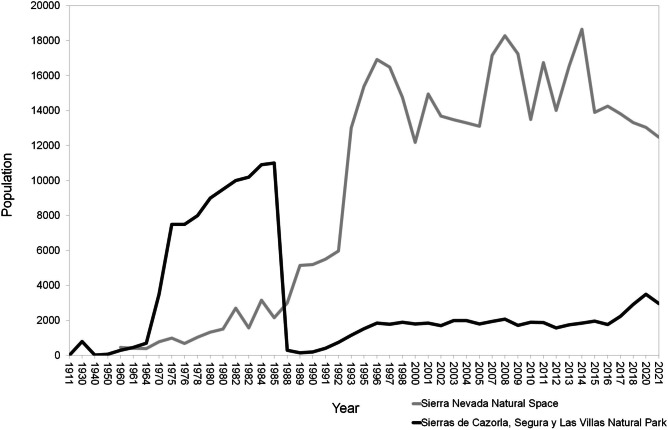
Different population trends in two Iberian ibex populations affected by sarcoptic mange, namely, Sierras de Cazorla, Segura y Las Villas Natural Park population (deep gray) and Sierra Nevada Natural Space population (light gray), before and after the outbreak in 1985-1986 and 1992-1993, respectively

Beyond these particular determinants, in naïve free-ranging populations of wild mountain ungulates, the entry of *S. scabiei* produces an epizootic of disease that spreads to affect the whole population in a variable number of years (Fernández-Morán et al. 1997; Pérez et al. 1997a; León-Vizcaíno et al. 1999; Rossi et al. 2007; Turchetto et al. 2014; Valldeperes et al. 2018a, b; Obber et al. 2022; Sosa et al. 2022). Such outbreaks are usually associated with variable mortality rates until sarcoptic mange becomes enzootic and the population eventually starts recovering. Nevertheless, the affected populations do not always reach the densities existing before the epizootics, which are also conditioned by other factors such as population abundance and genetic variability before the outbreak, interspecific competition, culling management strategy, and other population, environment, and management factors (Fandos 1991; Loison et al. 1996; Pérez et al. 1997a, 2021, 2022; León-Vizcaíno et al. 1999; González-Quirós et al. 2002a, b; González-Candela et al. 2004; Rossi et al. 2007; Nores and González-Quirós 2009; Espinosa et al. 2020; Granados et al. 2020) (Fig. 2).

**Fig. 3:**
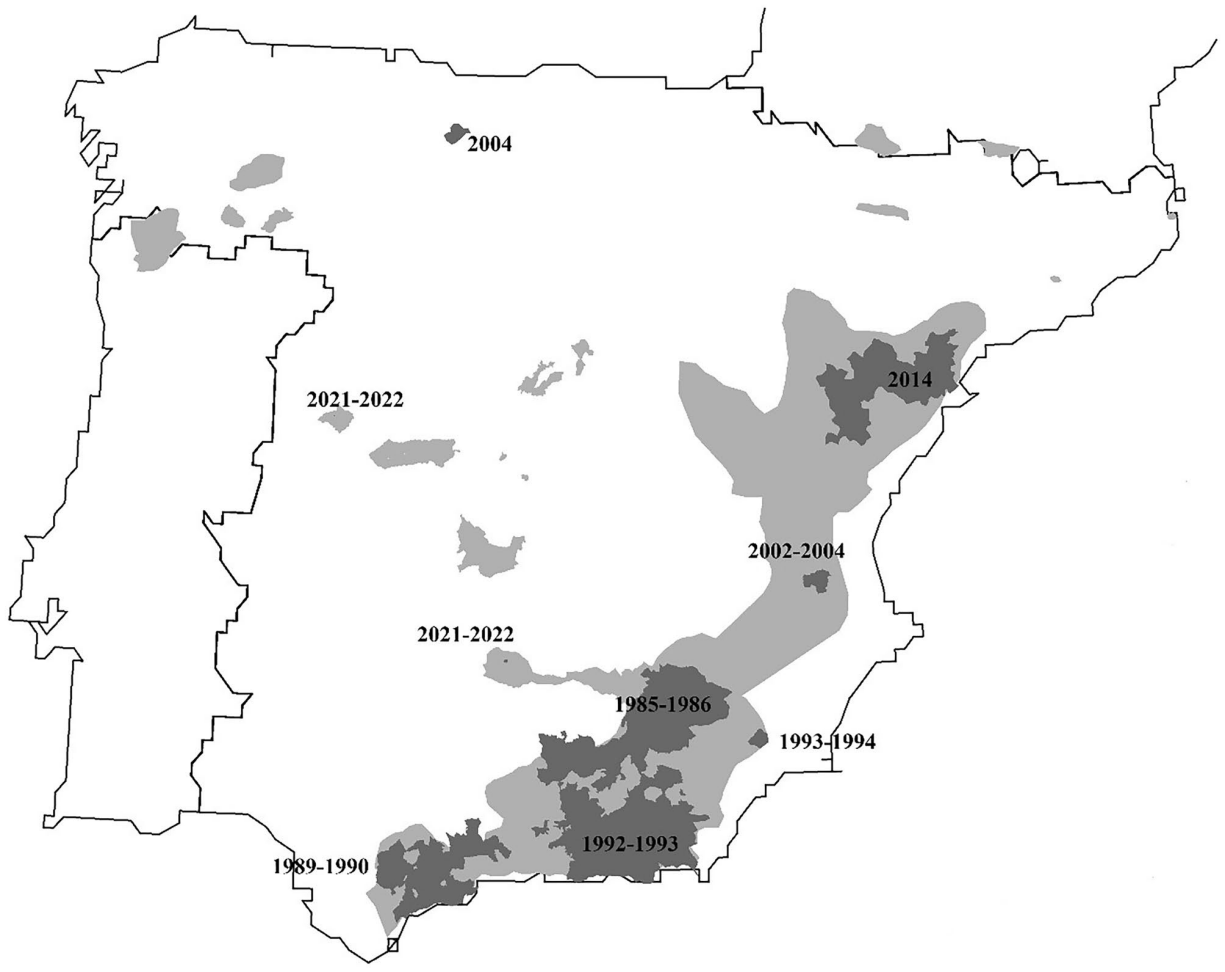
Distribution of Iberian ibex populations not affected (light grey) and affected (dark grey) by sarcoptic mange at a municipality scale, indicating the year(s) of the first description or initial outbreak of sarcoptic mange in each affected population

This eco-epidemiological transition from epizootic outbreak to endemic disease participating as a regulation factor in population dynamics has occurred in most of the Iberian ibex populations affected by sarcoptic mange (Fig. 3; Fandos 1991; Pérez et al. 1992; Pérez et al. 1994; Palomares and Ruiz-Martínez et al. 1993; Ruiz Martínez et al. 1996; Pérez et al. 1997a; Sánchez 1998; León-Vizcaino et al. 1999; Sánchez Isarria et al. 2008a, b; Prada and Herrero 2013; Mentaberre et al. 2015; Valldeperes et al. 2018a, b). The initial outbreaks provoked variable mortalities, reaching up to 90% (Fandos 1991; Pérez et al. 1997a; León-Vizcaíno et al. 1999), but afterward, the disease remained enzootic in all the affected populations, with prevalences even below 1% and, correspondingly, associated low mortality (Ruiz-Martínez et al. 1993). These enzootic situations, however, can be intercalated with sporadic outbreaks with associated mortality below 20% (Gil Collado 1960).

**Fig. 4:**
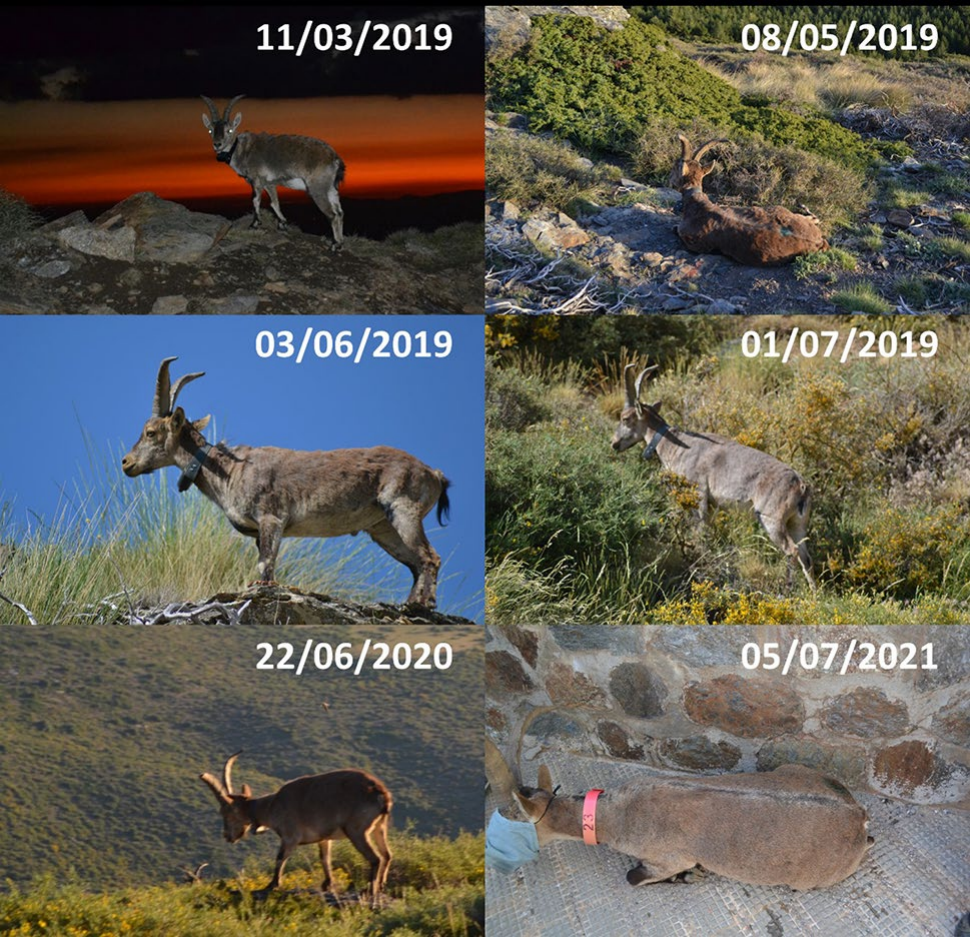
Male Iberian ibex captured and GPS-collared in Sierra Nevada Natural Space and monitored for more than 2 years. The disease evolved through different infestation stages, and finally, the ibex healed and completely recovered from sarcoptic mange spontaneously without treatment. Similar trends were observed in a number of individuals

Such eco-epidemiological transition can only be explained by the apparition of mange-resistant hosts during the epizootic phase (Guberti and Zamboni 2000; Turchetto et al. 2014; Pérez et al. 2022), as demonstrated, among other evidences, by (1) the transition of sarcoptic mange in the affected populations from epizootic to enzootic, with decreasing prevalences reaching values down to 1% (Fandos 1991; Pérez et al. 1997a, 2021, 2022; León-Vizcaíno et al. 1999; Valldeperes et al. 2018a, b; Granados et al. 2020); (2) the differential clinical outcome of free-ranging mangy ibexes, with recovery and long-term survival of the resistant individuals (over 649 days), as compared to critical and rapidly lethal clinical evolution in the non-resistant ones (around 90 days) (León-Vizcaíno et al. 1999; Alasaad et al. 2013) (Fig. 4); and (3) the identification of the same clinical trends and outcomes (total recover, partial recover, and terminal) in Iberian ibexes experimentally infested with *S. scabiei* (Ráez-Bravo et al. 2015, 2016; Espinosa et al. 2017d, 2017b; Ráez-Bravo 2019; Valldeperes et al. 2019).

#### Management of sarcoptic mange in Iberian ibex populations

The management of sarcoptic mange in wild Caprinae populations has been recently revised (Espinosa et al. 2020; Pérez et al. 2021). Briefly, potential measures include (1) preventing the entry of a disease in a naïve population; (2) eradicating a disease already present in a population; (3) controlling a disease present in a population, maintaining low prevalence but without eradicating it; or (4) not acting (Wobeser 1994, 2002; Espinosa et al. 2020). Any wildlife disease management, however, must be integrated with a comprehensive holistic management of the ecosystem (Aguirre et al. 1995).

1. Since domestic livestock is the origin of most of the outbreaks of sarcoptic mange in Iberian ibex (Lavín et al. 2000a; Moroni et al. 2021), controlling sarcoptic mange in sympatric small ruminant flocks seems therefore a key to control the entry of *S. scabiei* in naïve Iberian ibex populations.
2. Eradication of *S. scabiei* is deemed impossible and unfeasible even in human populations, where known treatments exist and all the individuals can be treated (Falk 1982; Arlian 1989). However, Iberian ibex depopulation has been proposed to eradicate sarcoptic mange in this species (Arenas, et al. 2002, 2009a, b), although it is against all odds and the minimum essential requirements of an integrated ecosystem management of wildlife diseases (Aguirre et al. 1995; Espinosa et al. 2020). Moreover, even if complete Iberian ibex depopulation was achieved, other sympatric mammal hosts, either domestic or wild, could maintain the mite, acting as a reservoir until an eventual repopulation (natural or anthropic) by Iberian ibex.
3. Ivermectin is an antiparasitic macrocyclic lactone traditionally used to control sarcoptic mange in domestic animals (Ibrahim and Abu-Samra 1987; Geary 2005), which has also been parenterally used to deworm wildlife species and boost the immune system (López-Olvera et al. 2006). The successful individual treatment of sarcoptic mange in captive Iberian ibexes with parenteral injections of ivermectin (Pérez et al. 1996c; León-Vizcaíno et al. 2001; Sarasa et al. 2010) has led to the empiric mass release of ivermectin-medicated feed in natural protected areas in eastern Spain inhabited by Iberian ibex populations affected by sarcoptic mange (Sánchez-Isarria et al. 2008a, b; Valldeperes et al. 2022a), being even legally authorized in different regions within Spain, such as Andalucía (southern Spain) and Aragon (northern Spain) (Junta de Andalucía 2021; Gobierno de Aragón 2022).

However, oral administration of macrocyclic lactones to free-ranging wildlife populations through medicated feed is mostly empiric and entails unanswered concerns regarding its usefulness, efficacy, and potential risks to the target species, other wildlife, the environment, and even human public health (Rowe et al. 2019; Espinosa et al. 2020; Moroni et al. 2020; Valldeperes et al. 2022b). On-field administration of in-feed ivermectin-induced mean plasma concentrations below the therapeutic threshold was reported for other parasites and only in a proportion of Iberian ibexes, which is insufficient to decrease *S. scabiei* transmission (Nolan et al. 1985; Pound et al. 1996; Davey et al. 2010; Valldeperes et al. 2022a). Moreover, ivermectin plasma concentration decreased below 20% in 72 h after experimental administration in Iberian ibexes (Moroni et al. 2022a), further demonstrating the lack of efficacy of this measure. On the other hand, massive administration of topic macrocyclic lactones can lead to overdosing (Old et al. 2021; Mounsey et al. 2022). Moreover, such inadequate dosage and massive release of antiparasitic drugs in the environment increase the probability of the mite generating resistance against the treatment (Tabashnik 1994; Vermunt 1996; Currie et al. 2004; Bliss et al. 2008; Terada et al. 2010; Andriantjoanirina et al. 2014), which adds to the detrimental environmental consequences for coprophagous arthropods and other non-target invertebrates (Sommer et al. 1992; Herd et al. 1996; Verdú et al. 2015, 2018).

Conversely, monitoring and exceptional individual culling because of animal welfare only in severely affected ibexes have been implemented as a conservative and minimally interventionist management measure in Iberian ibex populations affected by sarcoptic mange (Ruiz-Martínez et al. 1996; Espinosa et al. 2020; Moroni et al. 2020). Such an approach eases the eco-epidemiological transition of mange from epizootic to enzootic, through the selection and survival of resistant host and mutual adaption of host and parasite (Granados et al. 2011). Besides, the simultaneous monitoring of population and health status (Cardoso et al. 2021; Barroso et al. 2022) allows for assessing, determining, and monitoring the prevalence and population trends over time (Espinosa et al. 2020; Granados et al. 2020; Pérez et al. 2021).

### Infectious keratoconjunctivitis

#### Etiology

Infectious keratoconjunctivitis is a disease affecting the eyes of domestic and wild small ruminants. The causal role of keratoconjunctivitis has been attributed to a number of bacteria, including *Corynebacterium pyogenes*, *Escherichia coli*, *Chlamydia psittaci*, *Ch. abortus*, *Moraxella bovoculi*, *Moraxella* (*Branhamella*) *ovis* (formerly named *Neisseria ovis*), *Listeria monocytogenes*, *Mycoplasma* other than *M. conjunctivae* (including *M. agalactiae*), *Staphylococcus aureus*, or *Rickettsia*-like spp. (Bijlenga et al. 1983; Blanco et al. 1982; Egwu et al. 1989; Dagnall 1994a, b; Rodríguez et al. 1996; González-Candela et al. 2007; Verbisck-Bucker et al. 2008; Verbisck et al. 2010; Holzwarth et al. 2011; Arnal et al. 2013; Osman et al. 2013; Dickey et al. 2016). However, evidences have finally identified that infectious keratoconjunctivitis is related to the infection by virulent strains of *M. conjunctivae* (Nicolet and Freundt 1975; Giacometti et al. 1998, 1999; Zimmermann et al. 2008), although *M. conjunctivae* and *M. agalactiae* have been pointed out indistinctively as etiological agents in the past since both were phylogenetically classified together (Rodríguez et al. 1996; González-Candela et al. 2007, León-Vizcaíno et al. 2008c; Verbisck-Bucker et al. 2008; Verbisck et al. 2010). Initially, *M. conjunctivae* infection was believed to always cause clinical disease in wild mountain ungulate populations (Giacometti et al. 1998, 1999, 2002a, b), but later studies showed that *M. conjunctivae* can be carried out asymptomatically by these species (Ryser-Degiorgis et al. 2009; Mavrot et al. 2012; Fernández-Aguilar et al. 2017a, b). Nevertheless, the role of *Chlamydiaceae* in infectious keratoconjunctivitis of wild free-ranging mountain ungulate populations is still in discussion (Holzwarth et al. 2011; Arnal et al. 2013; Dias-Alves et al. 2021).

#### Symptomatology

**Fig. 5:**
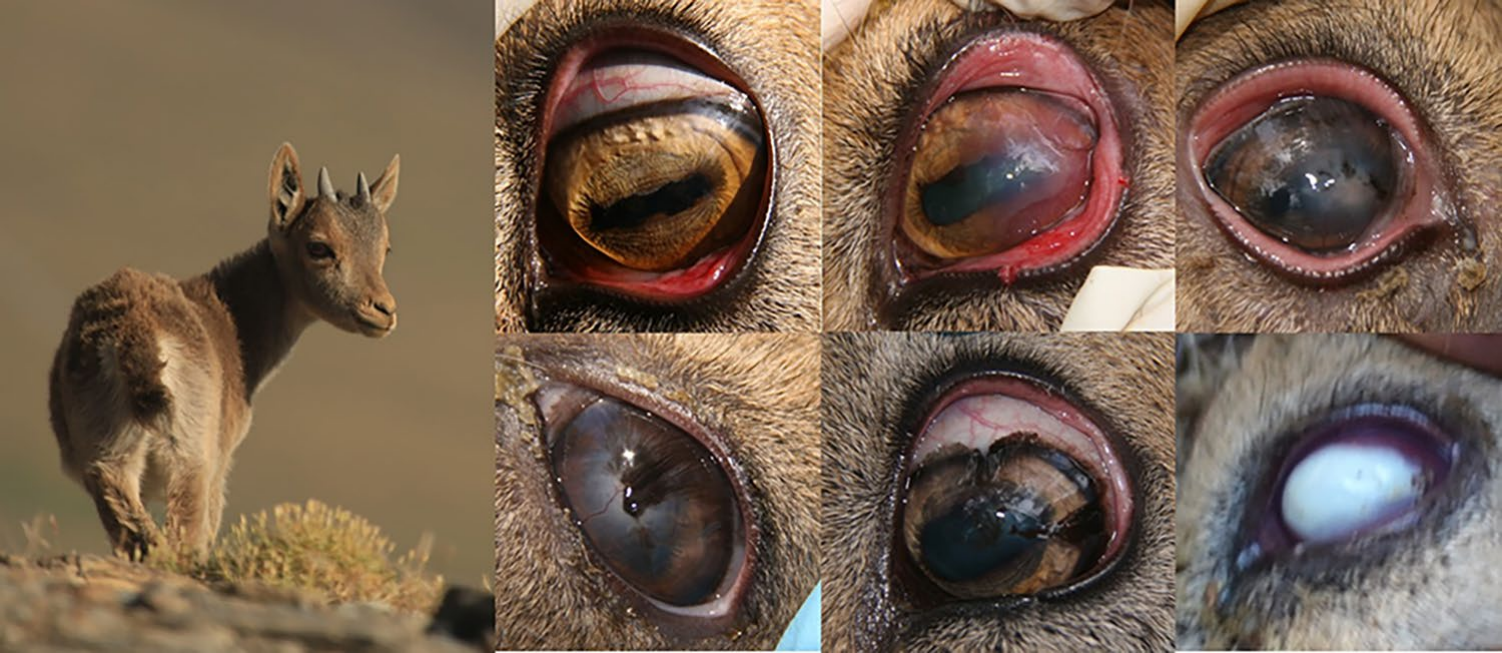
Free-ranging Iberian ibex kid from Sierra Nevada Natural Space with mild conjunctivitis in the right eye consistent with infectious keratoconjunctivitis. Different clinical stages of infectious keratoconjunctivitis caused by *Mycoplasma conjunctivae* in Iberian ibexes kept in captivity in Sierra Nevada (Espinosa et al. 2017c; Fernández-Aguilar et al. 2017b)

Clinical signs of infectious keratoconjunctivitis include blepharospasm, conjunctivitis, epiphora, varying degrees of corneal opacity and ulceration, ocular damage, and inflammation, which cause visual impairment and blindness (Mayer et al. 1997). The disease is usually transient, and the infection commonly courses to spontaneous clinical recovery, although clinical signs may progress to staphyloma and corneal perforation (Giacometti et al. 1998; Fernández-Aguilar et al. 2017a, b, c) (Fig. 5).

#### Diagnosis

In the past, the study of infectious keratoconjunctivitis was limited by the performance of traditional culture techniques, not only in wildlife but also in domestic animals, due to the difficulty to culture and isolate *M. conjunctivae* (Nicolet and Freundt 1975; Giacometti et al. 1998, 1999). However, the development of molecular techniques, namely, PCR, allowing the identification, characterization, and sequencing of *M. conjunctivae* has allowed not only to certify the central role of this pathogen in the etiology of infectious keratoconjunctivitis, but also to diagnose this disease in infected individuals and to understand the relationship between *M. conjunctivae* strains and disease (Vilei et al. 2007; Fernández-Aguilar. 2017a, b, c; Yang et al. 2022). Additionally, serological tests detecting antibodies both in serum and in lachrymal against *M. conjunctivae* allow retrospective epidemiological studies in wild free-ranging mountain ungulates populations (Belloy et al. 2001).

#### Epidemiology

*Mycoplasma conjunctivae* is transmitted through direct contact and by flies, which act as mechanical vectors (Degiorgis et al. 1999; Giacometti et al. 2002a; Fernández-Aguilar et al. 2019), allowing infectious keratoconjunctivitis to spread at a speed estimated over 15 km/year (Degiorgis et al. 2000). This disease is characterized by a relatively high morbidity with a low mortality ranging from 5% up to 27%, mostly due to starvation or falls in the steep mountain environment inhabited by wild caprines during the blindness stage of the disease (Loison et al. 1996; Degiorgis et al. 2000; Giacometti et al. 2002a). Mountain ungulate populations can be demographically affected both by keratoconjunctivitis epidemics and the associated culling management to try to reduce density to decrease *M. conjunctivae* transmission, but populations usually recover in 5 years after the outbreak (Gauthier 1991; Loison et al. 1996). Clinical infectious keratoconjunctivitis follows a seasonal pattern, with more cases in summer due to the increase of susceptible host densities, because of the birth of naïve kids and the higher abundance of insect vectors of *M. conjunctivae*. Although the morbidity is lower in winter, the relative mortality is higher due to the more adverse environmental conditions (Rossi et al. 2019). Small domestic ruminants, mainly sheep, were traditionally assumed as the main reservoir of *M. conjunctivae*, and wild mountain caprines were considered spillover hosts that could not maintain infectious keratoconjunctivitis without the participation of domestic hosts (Giacometti et al. 2002a, b; Fernández-Aguilar et al. 2013, 2017c; Rossi et al. 2019). However, separate independent epidemiological cycles in domestic and wild ruminants and the capability of wild mountain ungulate populations to maintain *M. conjunctivae* circulation without the participation of domestic hosts have been demonstrated later (Ryser-Degiorgis et al. 2009; Fernández-Aguilar et al. 2017a, b).

#### Infectious keratoconjunctivitis in Iberian ibex populations

In Iberian ibex, *M. conjunctivae* has been reported in association with sporadic cases of keratoconjunctivitis (Antón et al. 1999; Cubero et al. 2002; Arnal et al. 2009; Revilla Calavia 2012), outbreaks in free-ranging and captive populations (León-Vizcaíno et al. 2008c; Fernández-Aguilar et al. 2017b), and endemicity in free-ranging populations (Ramírez-Rosales 2018) (Fig. 5, Table 1). While prevalences can be low in unaffected Iberian ibex populations, without the presence of *M. conjunctivae* in asymptomatic individuals (León-Vizcaíno et al. 2008c; Revilla Calavia 2012; Fernández-Aguilar et al. 2017b), they can rise during epizootics and reach even higher enzootic values after the epizootic, turning to asymptomatic as in domestic small ruminant flocks (Fernández-Aguilar et al. 2013, 2017b, c; Ramírez-Rosales 2018) (Table 1). The 1-year evolution from an outbreak with severe clinical signs to an asymptomatic endemic status in a captive Iberian ibex population (Fernández-Aguilar et al. 2017b) suggests that at least certain outbreaks can be related to the exposure of populations to new virulent strains of *M. conjunctivae*, until the relationship between the pathogen and the host ibex population evolves to an endemic balance situation. This finding is consistent with the higher prevalence of *M. conjunctivae* described in young individuals, which later achieve to eliminate or control the infection as they develop an efficient immune response with increasing age and exposure to the infectious agent (Giacometti et al. 2002a, b; González-Candela et al. 2006, 2007; León-Vizcaíno et al. 2008c; Fernández-Aguilar et al. 2017a, b, c).

**Table 1:** Prevalences and 95% central confidence intervals reported for *Mycoplasma conjunctivae*, *Corynebacterium pseudotuberculosis*, *Oestrus caucasicus*, and *Babesia ovis* in Iberian ibex

**Reference**	**Prevalence**	**95% central CI (%)**	**Sample**	**Technique**	**Comments**
Infectious keratoconjunctivitis (*Mycoplasma conjunctivae*)					
Antón et al. (1999)	0.4%			Culture	No further data available in the reference
Cubero et al. (2002)	0.4% (2/450)	0.0–1.1	Serum	CFT	
González-Candela et al. (2006, 2007)	14.3% (46/321)	10.5–18.2	Conjunctive/ear swabs	Culture	*Mycoplasma agalactiae*
León Vizcaíno et al. (2008c)	0.8% (2/259)	0.0–1.8	Conjunctive swabs	Culture	
	0.48% (2/415)	0.0–1.1	Serum	ELISA	
Arnal et al. (2009)	27.8% (5/18)	7.1–48.5	Conjunctive swabs	PCR	All the ibexes sampled had clinical signs
Revilla Calavia (2012)	3.6% (7/196)	1.0–6.2	Conjunctive swabs	PCR	29/196 of the ibexes sampled had clinical signs
	24.1% (7/29)	8.6–39.7			All the ibexes sampled had clinical signs
	0.0% (0/167)	0.0–0.0			None of the ibexes sampled had clinical signs
Fernández-Aguilar et al. (2017b)	35.4% (17/48)	23.4–49.6	Conjunctive swabs	PCR	Epizootic in a captive population
	8.7% (12/46)	3.4–20.3			Post-epizootic decrease in a captive population
	4.3% (2/46)	1.2–14.5			Post-epizootic decrease in a captive population
	75.3% (52/69)	64.0–84.0			Asymptomatic enzootic in a captive population
	68.7% (33/48)	54.7–80.0	Serum	ELISA	Epizootic in a captive population
	54.3% (25/46)	40.2–67.8			Post-epizootic decrease in a captive population
	46.5% (20/43)	32.5–61.1			Post-epizootic decrease in a captive population
	38.5% (10/26)	23.4–59.3			Asymptomatic enzootic in a captive population
Ramírez-Rosales (2018)	17.7% (26/147)	11.9–24.8	Conjunctive swabs	PCR	Enzootic in a free-ranging population
Caseous lymphadenitis (*Corynebacterium pseudotuberculosis*)					
González-Candela et al. (2006)	3.1% (10/321)	1.2–5.0	Conjunctive, nasal, and vaginal swabs	Culture	*Corynebacterium* sp.
León Vizcaíno et al. (2008b)	2.9% (1/35)	0.0–8.4	Serum	ELISA	
Colom-Cadena et al. (2014)	0.0% (0/18)	0.0–0.0	Inspection	Lesions	Initial entry at an enclosure from the wild
	68.8% (11/16)	46.0–91.5			Epizootic after less than 1 year in captivity
	5.6% (1/18)	0.0–16.1	Serum	ELISA	Initial entry at an enclosure from the wild
	85.7% (12/14)	67.4–100.0			Epizootic after less than 1 year in captivity
Varela-Castro et al. (2017)	18.9% (68/360)	14.8–22.9	Serum	ELISA	Enzootic in a free-ranging population
*Oestrus caucasicus*					
Pérez et al. (1996a, b)	73.9% (133/180)	67.5–80.3	Head	Sinus inspection	
Pérez et al. (1997b)	67.1% (271/404)	62.5–71.7	Head	Sinus inspection	
Antón et al. (2002)	59.0% (343/581)	55.0–63.0	Head	Sinus inspection	
Calero-Bernal et al. (2020)	9.5% (4/42)	0.6–18.4	Serum	ELISA	Antibodies against *Oestrus* sp.
*Babesia ovis*					
Ferrer et al. (1998)	32.6% (155/475)	28.4–36.8	Serum	IFAT	
Antón et al. (2002)	59.8% (73/122)	51.1–68.5	Serum	IFAT	
	0.0% (0/110)	0.0–0.0	Blood smear	Microscopic inspection	
Calero-Bernal et al. (2020)	43.4% (56/129)	34.9–52.0	Serum	IFAT	

*CFT* complement fixation test, *CI* confidence interval, *ELISA* enzyme-linked immunoassay, *IFAT* indirect fluorescent antibody test, *PCR* polymerase chain reaction

#### Management of infectious keratoconjunctivitis

Targeted health surveillance for *M. conjunctivae* in transhumant domestic small ruminant flocks (Lawson et al. 2021) should help to prevent the introduction of new virulent strains of *M. conjunctivae* in Iberian ibex populations, although this will not prevent the circulation of the strains already endemically maintained in the wildlife populations (Fernández-Aguilar et al. 2013, 2017a, c; Rossi et al. 2019).

### Caseous lymphadenitis

#### Etiology

Caseous lymphadenitis is a chronic infectious disease caused by the bacterium *Corynebacterium pseudotuberculosis*, affecting domestic and wild small ruminants (Dorella et al. 2006; Domenis et al. 2018).

#### Pathogenesis and symptomatology

**Fig. 6:**
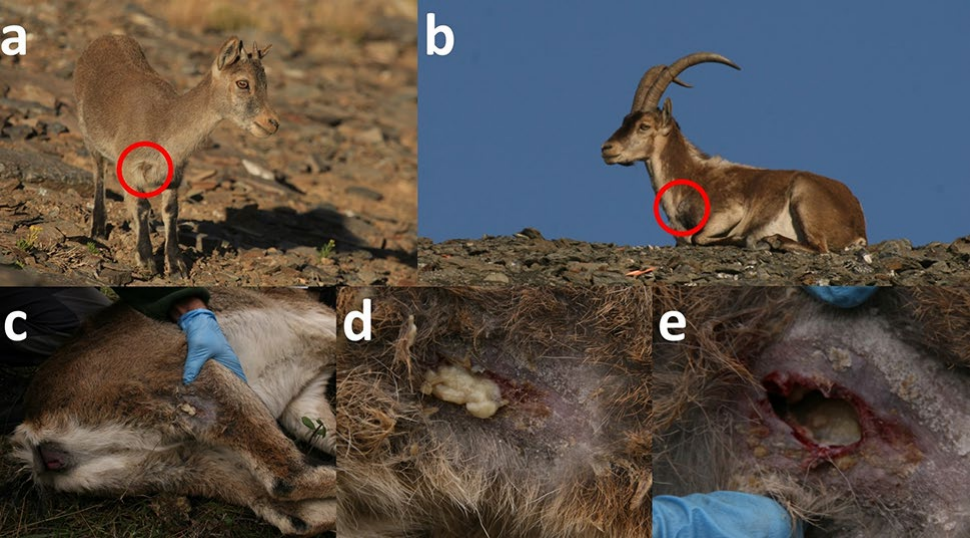
Free-ranging Iberian ibex kid (**a**) and adult male (**b**) from Sierra Nevada with apparently draining prescapular lymph node fistulae, compatible with *Corynebacterium pseudotuberculosis* infection. **c**–**e** Fistulized popliteal lymph node of a female Iberian ibex kept in captivity at the Ports de Tortosa i Beseit National Game Reserve enclosure. The presence of *C. pseudotuberculosis* was confirmed through bacterial culture and microbiological characterization (Colom-Cadena et al. 2014; Espinosa et al. 2017c)

This disease is characterized by caseous lesions most frequently affecting the superficial lymph nodes, which can open through fistulae through the skin and leak their necrotic-purulent content (Fig. 6). The lesions may also affect parenchymal organs, and then, the disease can often be fatal, with progressive cachexia, weakness, and death (Baird and Fontaine 2007; Domenis et al. 2018).

#### Diagnosis

The diagnosis is based on compatible clinical signs and lesions and is further confirmed by bacteriological culture and identification or PCR from the purulent exudates (Colom-Cadena et al. 2014; Espinosa et al. 2017c). Antibodies against *C. pseudotuberculosis* can also be investigated through ELISA tests, allowing retrospective epidemiological studies at the population scale (Solanet et al. 2011; Colom-Cadena et al. 2014; Varela-Castro et al. 2017).

#### Caseous lymphadenitis in Iberian ibex

*Corynebacterium pseudotuberculosis* has been reported in Iberian ibex, both endemically in free-ranging populations and associated with disease outbreaks with mortality due to visceral forms in captive populations (González-Candela et al. 2006; León-Vizcaíno et al. 2008b; Colom-Cadena et al. 2014; Varela-Castro et al. 2017) (Fig. 6, Table 1), but there is a lack of knowledge on the potential demographic effects of this disease on the populations of this species. Although domestic livestock has been suspected as the origin of caseous lymphadenitis in the affected Iberian ibex populations, once the interspecific transmission has occurred, *C. pseudotuberculosis* can circulate and probably be maintained by Iberian ibex alone, both in captive and free-ranging populations (Colom-Cadena et al. 2014; Varela-Castro et al. 2017). Captivity seems to enhance the transmission of *C. pseudotuberculosis* and the severity of the clinical signs due to increased pathogen circulation, increased risk of penetrating wounds caused by the facilities or in intraspecific fights, and immunosuppression induced by prolonged captivity-related stress (Colom-Cadena et al. 2014; Espinosa et al. 2017c). Although endemic caseous lymphadenitis probably has little relevance in Iberian ibex free-ranging populations, it can hinder the management of captive populations or in intensive game estates aimed at conservation actions or game resource exploitation, respectively (Granados et al. 1996b; Colom-Cadena et al. 2014; Espinosa et al. 2017c).

#### Management of caseous lymphadenitis in Iberian ibex populations

In captivity, individual treatment of the affected ibexes through aspiration, cleaning, and disinfection of the purulent material and fistulae and administration of systemic antibiotic, combined with autovaccination using the strains isolated in the population, have been recommended and have apparently successfully controlled outbreaks (Colom-Cadena et al. 2014; Espinosa et al. 2017c). Since the epidemiological knowledge of *C. pseudotuberculosis* in Iberian ibex is still limited, no specific management recommendations exist for the management of caseous lymphadenitis in free-ranging Iberian ibex populations, apart from identifying the epidemiological status of the population and the potential domestic and wild reservoirs participating in the circulation and maintenance of the pathogen (Varela-Castro et al. 2017).

### Parasitic diseases

Most of the parasites identified in Iberian ibex are shared with domestic livestock (Rossi et al. 1994; Pérez et al. 1996c) and are not related to clinical disease (Calero-Bernal et al. 2020). However, some parasites are exclusive or at least more prevalent in wild ungulates and specifically in Iberian ibex than in domestic animals. This seems to be the case for the larvae of the Diptera *Oestrus* spp., with prevalences above 87% in the Iberian ibex population in Sierra Nevada, usually parasitizing nasal, sinus, and horn cavities (Ruiz-Martínez et al. 1992; Granados et al. 1996a; Pérez et al. 1996b, 1997b; Antón et al. 2002) (Table 1). Another parasite apparently common and frequently reported in Iberian ibex populations is the intracellular protozoan *Babesia ovis*. Not only a contact with this parasite has been reported in Iberian ibex through serology (Ferrer et al. 1998; Calero-Bernal et al. 2020) (Table 1), but mortality related to babesiosis has also been described in a Mediterranean Iberian ibex population (Marco et al. 2000). In this same population, the gastrointestinal nematode *Haemonchus contortus* also was reported to cause mortality in Iberian ibex, apparently related to a combination of high host population density with environmental conditions that favored the parasite (Lavín et al. 1997).

## Diseases under targeted surveillance in the Iberian ibex

Apart from the sarcoptic mange, the Spanish national wildlife health surveillance program includes brucellosis among the diseases subjected to targeted health surveillance (Lawson et al. 2021; Gobierno de España et al. 2022). Additionally, to move Iberian ibex among game estates and/or natural areas, the Spanish legislation requires performing specific diagnostic tests for tuberculosis and bluetongue, as well as for sarcoptic mange and brucellosis as aforementioned (Gobierno de España et al. 2009). Although an impact of brucellosis, tuberculosis, and bluetongue on Iberian ibex population dynamics has not been reported, brucellosis and tuberculosis are zoonotic diseases shared with livestock subjected to eradication programs (Gobierno de España et al. 1996; Gobierno de España 2003), and bluetongue is a notifiable animal disease (Gobierno de España et al. 2014), which motivates the targeted surveillance of these three diseases for Iberian ibex movements in Spain (Gobierno de España et al. 2009; Lawson et al. 2021).

### Brucellosis

Brucellosis is a zoonotic disease caused by the bacterium *Brucella melitensis* and *B. abortus*, which can affect domestic and wild animals and humans (Corbel 1997; Seleem et al. 2010). Despite being included in the targeted health surveillance of Iberian ibex (Gobierno de España et al. 2009, 2022), a single clinical case with isolation of *B. melitensis* biovar 1 (Muñoz et al. 2010) has been reported, and the seroprevalences detected in this species range from 0 to 2.6% depending on the diagnostic test used. This suggests that Iberian ibex is not a reservoir for brucellosis but a spillover accidental host (Antón et al. 1999; León-Vizcaíno et al. 2008a; Muñoz et al. 2010; Astorga-Márquez et al. 2014; Junta de Andalucía 2015; Calero-Bernal et al. 2020; Gómez-Guillamón et al. 2020) (Table 2). Nevertheless, the isolation of *B. melitensis* in the closely related Alpine ibex (*Capra ibex*) was first reported almost 25 years ago (Ferroglio et al. 1998), and this species has been described as the first wild ungulate species maintaining and possibly acting as a reservoir for brucellosis in the last decade, causing serious concern because of spillback to domestic cattle (Hars et al. 2013, 2015; Rossi et al. 2019). Since the causes for the new reservoir role for brucellosis in this species and the management of this disease are still unclear and generate concern and conflict (Lambert et al. 2020, 2021, 2022), keeping brucellosis surveillance and preventing the entry of this disease in Iberian ibex populations are warranted.

**Table 2:** Prevalences and 95% central confidence intervals reported for brucellosis, tuberculosis, and bluetongue in Iberian ibex

**Reference**	**Prevalence**	**95% central CI (%)**	**Sample**	**Technique**
Brucellosis (*Brucella melitensis* and *B. abortus*)				
Antón et al. (1999)*	1.0%			Culture
Cubero et al. (2002)	0.9% (4/450)	0.0–1.8	Serum	RBT, CFT
León Vizcaíno et al. (2008a)	1.0% (6/598)	0.2–1.8	Serum, lymph node, spleen, vaginal fluid	Culture, PCR, RBT, CFT, ELISA
Muñoz et al. (2010)	0.3% (3/1086)	0.0–0.6	Serum	ELISA
Revilla Calavia (2012)	0.0% (0/1474)	0.0–0.0	Serum	ELISA
Astorga Márquez et al. (2014)	0.0% (0/214)	0.0–0.0	Serum	RBT, CFT
Junta de Andalucía (2015)	2.6% (15/571)	1.3–3.9	Serum	RBT
	0.3% (2/585)	0.0–0.8		CFT
Calero-Bernal et al. (2020)	0.0% (0/50)	0.0–0.0	Serum	RBT, CFT, AGIDT
	0.0% (0/50)	0.0–0.0	Genital mucosa	Culture
Gómez-Guillamón et al. (2020)	2.5% (2/549)	1.2–3.8	Serum	RBT
	0.4% (2/564)	0.0–0.8		CFT
Tuberculosis (*Mycobacterium tuberculosis* complex)				
Cubero et al. (2002)	0.0% (0/175)	0.0–0.0	Lung, lymph node	Immunofluorescence
León Vizcaíno et al. (2008b)	0.0% (0/35)	0.0–0.0	Carcass	ELISA, lesions, culture
Mentaberre et al. (2010)	0.0% (0/205)	0.0–0.0	Carcass	Lesions
Revilla Calavia (2012)	1.5% (10/648)	0.6–2.5	Serum	ELISA
	0.0% (0/150)	0.0–0.0	Carcass	Necropsy, culture, Ziehl–Neelsen
García-Bocanegra et al. (2012a)	0.0% (0/460)	0.0–0.0	Serum	ELISA
Mentaberre et al. (2014)	0.0% (0/355)	0.0–0.0	Carcass	Lesions
Ruiz-Rodríguez (2018)	14.6% (35/239)	10.2–19.1	Serum	ELISA
Calero-Bernal et al. (2020)	6.0% (3/50)	0.0–12.6	Serum	ELISA
	0.0% (0/50)	0.0–0.0	Lung, mediastinal lymph nodes	Culture
González-Insa (2021)	7.9% (3/38)	0.0–16.5	Serum	ELISA
Bluetongue virus				
García et al. (2009)	10.8% (9/83)	0.0–32.4	Serum	ELISA
Lorca-Oró et al. (2011)	4.0% (31/770)	2.6–5.4	Serum	ELISA
	0.0% (0/380)	0.0–0.0	Blood	PCR
	2.9% (1/34)	0.0–8.6	Spleen	PCR
Revilla Calavia (2012)	0.3% (2/722)	0.0–0.7	Serum	ELISA
Astorga Márquez et al. (2014)	1.9% (4/214)	0.1–3.7	Serum	ELISA
Lorca-Oró et al. (2014)	11.1% (4/36)	0.8–21.4	Serum	ELISA
Junta de Andalucía (2015)	3.4% (19/559)	1.9–4.9	Serum	ELISA
Gómez-Guillamón et al. (2020)	3.3% (18/538)	1.8–4.9	Serum	ELISA

*AGID* agar gel immunodiffusion test, *CFT* complement fixation test, *ELISA* enzyme-linked immunoassay, *PCR* polymerase chain reaction, *RBT* Rose Bengal test

^*^No further data available in this reference

### Tuberculosis

Tuberculosis is another zoonosis in the wildlife-livestock-human interface, caused by bacteria of the *Mycobacterium tuberculosis* complex (MTC) (Desta et al. 2022). A single clinical case of apparent clinical tuberculosis attributed to *Mycobacterium bovis* affecting the lung and liver in a female Iberian ibex has been reported out of a sample size of 450 (0.2%), although the confirmation through microbiological culture diagnostic techniques was not specified. This same study did not achieve to identify *M. bovis* through immunofluorescence in the lungs and lymph nodes of 175 Iberian ibexes (Cubero et al. 2002). Antibodies against MTC have been detected in Iberian ibex with seroprevalences up to 14.6% (Revilla Calavia 2012; Ruiz-Rodríguez 2018; Calero-Bernal et al. 2020) (Table 2). However, clinical cases of tuberculosis-like lesions confirmed through microbiological culture have not been described in the available literature or official reports, not even in areas with a known circulation of MTC in the wildlife-livestock interface and with apparent high infection pressure (León Vizcaíno et al. 2008b; Mentaberre et al. 2010, 2014; García-Bocanegra et al. 2012a; Revilla Calavia 2012) (Table 2). Hence, resistance against tuberculosis, either behavioral, immunological, and/or ecological, has been suggested for Iberian ibex (Mentaberre et al. 2010). Experimental studies including the challenge of Iberian ibex with MTC under controlled conditions should be carried out in order to elucidate whether this apparent resistance of Iberian ibex exists or not and whether it is related to ecological, behavioral, and/or immunological factors in case of existing. The results of such research should consequently justify the need to continue targeted surveillance of tuberculosis in this species or, conversely, allow the elimination of tuberculosis from the list of diseases to monitor and control in Iberian ibex.

### Bluetongue

Bluetongue is a disease caused by different serotypes of orbivirus, mainly transmitted by vector midges of the genus *Culicoides* (Mellor and Wittmann 2002; Mertens et al. 2004). This disease can affect domestic and wild ruminants and camelids and is a notifiable disease included in the health surveillance and eradication programs of the European Union (Council Directive 2000/75/EC). Consequently, bluetongue has been added to the diseases under surveillance previously to Iberian ibex translocations according to the specific Spanish legislation (Gobierno de España et al. 2009). Experimental infection has demonstrated that Iberian ibex is susceptible to bluetongue virus infection, albeit subclinically (Lorca-Oró et al. 2012), and seroprevalences against bluetongue virus ranging from 3.4 to 11.1% have been reported in Iberian ibex free-ranging populations without associated clinical signs (García et al. 2009; Lorca-Oró et al. 2011; Astorga-Márquez et al. 2014; Lorca-Oró et al. 2014; Junta de Andalucía 2015; Gómez-Guillamón et al. 2020) (Table 2). Nevertheless, in 2018 the circulation of bluetongue virus serotype 4 in domestic ruminants was associated with an outbreak of mortality in sympatric Iberian ibexes (Gómez-Guillamón et al. 2021). The change in the epidemiological consideration of Iberian ibex with regard to bluetongue virus from susceptible asymptomatic host to clinical host where mortality can occur warrants further monitoring and targeting this disease in the health surveillance of this species.

In summary, except for sarcoptic mange, no significant epidemiological impact or demographic effect has been reported up to date for the remaining diseases under targeted health surveillance in Iberian ibex (brucellosis, tuberculosis, and bluetongue). Hence, the reason to monitor and survey these three diseases in Iberian ibex is rather their relevance in domestic livestock, where they are included in eradication programs, and the risk for zoonosis and transmission among domestic animals and wildlife from a One Health approach (Gortázar et al. 2007, 2016; Espinosa et al. 2017c; Rossi et al. 2019).

## Other diseases

Diseases not included in the targeted health surveillance of Iberian ibex and without known demographic effects have nonetheless been investigated in the populations of this species.

### Paratuberculosis

Paratuberculosis is an intestinal chronic wasting disease affecting ruminants, caused by the facultative intracellular pathogen *Mycobacterium avium* subspecies *paratuberculosis* (MAP) (Windsor 2015). MAP is considered endemic in Spain (Fanelli et al. 2020), where antibodies against MAP have been detected in wild ungulates (Reyes-García et al. 2008; López-Olvera et al. 2009; Falconi et al. 2010). Although the low prevalences generally found suggest that wild ungulates are not a reservoir for MAP (Carta et al. 2012, 2013), paratuberculosis outbreaks with associated mortality have been reported in naïve wild ungulate populations including Alpine ibex, but not in Iberian ibex (Marco et al. 2002; Ferroglio et al. 2000). Antibodies against MAP have been reported in this species with prevalences ranging from 0% up to 4.4% (Revilla Calavia 2012; Astorga-Márquez et al. 2014; Pizzato 2017; Ruiz-Rodríguez 2018; Calero-Bernal et al. 2020; Gómez-Guillamón et al. 2020) (Table 3). The spatial analyses of the seroprevalences obtained have pointed out domestic livestock as a risk factor for positivity in Iberian ibex, suggesting that this species is rather a spillover or dead-end host than a reservoir for MAP (Pizzato 2017; Ruiz-Rodríguez 2018). However, the detection of MAP by PCR in the ileocecal valve of 22% of the Iberian ibexes analyzed compared to the lower seroprevalence of 4.4% in the same population (Pizzato 2017) suggests that MAP carriage in Iberian ibexes could be underestimated when assessed through serological studies. Apart from the higher sensitivity of ileocecal valve PCR to diagnose MAP infection as compared to serum ELISA analysis in Iberian ibex (Pizzato 2017), these results raise a concern about a potential hidden role of this species in the epidemiology of MAP.

**Table 3:** Prevalences and 95% central confidence intervals reported for paratuberculosis, Crimean-Congo hemorrhagic fever virus, and abortive agents in Iberian ibex

**Reference**	**Prevalence**	**95% central CI (%)**	**Sample**	**Technique**	**Comments**
Paratuberculosis (*Mycobacterium avium* subsp. *paratuberculosis*)					
Cubero et al. (2002)	0.0% (0/5)	0.0–0.0	Intestine and lymph node	IF	
Revilla Calavia (2012)	1.1% (7/652)	0.3–1.9	Serum	ELISA	
Astorga Márquez et al. (2014)	1.9% (4/214)	0.1–3.7	Serum	ELISA	
Pizzato (2017)	9.2% (2/46)	6.2–12.3	Serum	ELISA	
	22.0% (20/91)	13.5–30.5	Ileocecal valve	PCR	
Ruiz-Rodríguez (2018)	4.4% (12/271)	2.0–6.9	Serum	ELISA	
Calero-Bernal et al. (2020)	0.0% (0/50)	0.0–0.0	Serum	ELISA	
	0.0% (0/50)	0.0–0.0	Ileocecal valve	Culture	
Gómez-Guillamón et al. (2020)	0.5% (3/564)	0.0–1.1	Serum	ELISA	
González-Insa (2021)	2.6% (1/38)	0.0–7.7	Serum	ELISA	
Crimean-Congo hemorrhagic fever virus (CCHFV)					
Espunyes et al. (2021)	78.6% (66/84)	69.8–87.3	Serum	ELISA	An Iberian ibex free-ranging population had a 100% prevalence of antibodies against CCHFV
Carrera-Faja et al. (2022)	96.0% (121/126)	92.6–99.4	Serum	ELISA	
Abortive agents					
*Chlamydophila* spp.					
Cubero et al. (2002)	13.3% (60/450)	10.2–16.5	Serum	CFT	*Chlamydia psittacci*
León Vizcaíno et al. (2008d)	13.4% (67/500)	10.4–16.4	Serum	ELISA	*Chlamydophila abortus*
Astorga Márquez et al. (2014)	0.0% (0/214)	0.0–0.0	Serum	ELISA	*Chlamydophila abortus*
Junta de Andalucía (2015)	0.0% (0/58)	0.0–0.0	Serum	ELISA	*Chlamydia abortus*
Varela-Castro et al. (2018)	30.0% (39/130)	22.1–37.9	Serum	ELISA	
	9.8% (11/112)	4.3–15.3	Spleen/Submandibular LN	PCR	
Calero-Bernal et al. (2020)	4.0% (2/50)	0.0–9.4	Serum	CFT	*Chlamydia* sp.
González-Insa (2021)	0.0% (0/38)	0.0–0.0	Serum	ELISA	*Chlamydophila abortus*
Q fever (*Coxiella burnetii*)					
León Vizcaíno et al. (2008d)	5.4% (27/500)	3.4–7.4	Serum	CFT	
Astorga Márquez et al. (2014)	12.6% (27/214)	8.2–17.1	Serum	ELISA	
Ruiz-Rodríguez (2018)	13.4% (34/254)	9.2–17.6	Serum	ELISA	
Varela-Castro et al. (2018)	30.0% (39/130)	10.0–29.8	Serum	ELISA	
	9.8% (11/112)	7.6–27.25	Spleen/Lymph node	PCR	
Calero-Bernal et al. (2020)	10.0% (5/50)	1.7–18.3	Serum	ELISA	
González-Insa (2021)	2.6% (1/38)	0.0–7.7	Serum	ELISA	
*Leptospira pomona*					
León Vizcaíno et al. (2008d)	0.0% (0/450)	0.0–0.0	Serum	MAT	
Contagious agalactia (*Mycoplasma agalactiae*, *M. mycoides* subsp. *mycoides*, and other mycoplasmas)					
Cubero et al. (2002)	2.0% (9/450)	0.7–3.3	Serum	CFT	*M. agalactiae*
González-Candela et al. (2007)	18.4% (59/321)	14.1–22.6	Conjunctive/ear swabs	Culture	All mycoplasmas
	14.3% (46/321)	10.5–18.2			*M. agalactiae*
	5.9% (19/321)	3.3–8.5			*M. arginini*
	0.3% (1/321)	0.0–0.9			*M. mycoides mycoides*
	6.2% (20/321)	3.6–8.9			Unidentified mycoplasmas
Verbisck-Bucker et al. (2008)	11.2% (46/411)	8.1–14.2	Conjunctive/ear swabs	Culture	*M. agalactiae*
Verbisck et al. (2010)	14.3% (5/35)	2.7–25.9	Conjunctive/ear swabs	Culture	*M. agalactiae*
	85.7% (30/35)	74.1–97.3	Serum	ELISA	
Astorga Márquez et al. (2014)	0.0% (0/214)	0.0–0.0	Serum	CFT	*M. mycoides mycoides*
	0.9% (2/214)	0.0–2.2	Serum	ELISA	*M. agalactiae*
Junta de Andalucía (2015)	5.8% (32/550)	3.9–7.8	Serum	ELISA	*M. agalactiae*
Gómez-Guillamón et al. (2020)	5.7% (30/529)	3.7–7.6	Serum	ELISA	*M. agalactiae*
*Neospora caninum*					
Almería et al. (2007)	0.0% (0/3)	0.0–0.0	Serum	ELISA	
León Vizcaíno et al. (2008d)	1.0% (5/485)	0.1–1.9	Serum	ELISA	
Calero-Bernal et al. (2020)	13.0% (14/108)	6.6–19.3	Serum	ELISA	
García-Bocanegra et al. (2012b)	5.6% (30/531)	3.7–7.6	Serum	ELISA	
	5.1% (27/531)	3.2–7.0	Serum	IFAT	
Cano-Manuel (2017)	4.8% (7/147)	1.3–8.2	Serum	ELISA	
	0.0% (0/147)	0.0–0.0	Serum	WB	
Border disease (pestivirus)					
León Vizcaíno et al. (2008d)	8.4% (42/500)	6.0–10.8	Serum	ELISA	
Astorga Márquez et al. (2014)	2.3% (5/214)	0.3–4.4	Serum	ELISA	
Junta de Andalucía (2015)	11.4% (62/545)	8.7–14.0	Serum	ELISA	
Gómez-Guillamón et al. (2020)	11.0% (58/525)	8.4–13.7	Serum	ELISA	
González-Insa (2021)	0.0% (0/38)	0.0–0.0	Serum	ELISA	
*Salmonella abortusovis*					
Cubero et al. (2002)	0.2% (1/450)	0.0–0.7	Serum	WAT	*S. abortusovis* serogroup B
León Vizcaíno et al. (2008d)	0.8% (4/500)	0.0–1.6	Serum	MAT	
Junta de Andalucía (2015)	0.4% (1/231)	0.0–1.3	Serum	ELISA	*Salmonella* sp.
*Toxoplasma gondii*					
León Vizcaíno et al. (2008d)	18.2% (91/500)	14.8–21.6	Serum	ELISA	
García-Bocanegra et al. (2012b)	27.5% (146/531)	23.7–31.3	Serum	MAT	
Cano-Manuel (2017)	11.6% (17/147)	6.4–16.7	Serum	ELISA	
	0.0% (0/147)	0.0–0.0	Serum	WB	
Calero-Bernal et al. (2020)	2.9% (4/137)	0.1–5.7	Serum	ELISA	
Almería et al. (2021)	13.9% (14/101)	7.4–20.6	Serum	MAT	
**Schmallenberg virus**					
Jiménez-Ruiz et al. (2021)	19.9% (49/246)	14.9–24.9	Serum	ELISA	
González-Insa (2021)	5.3% (2/38)	0.0–12.4	Serum	ELISA	

*CFT* complement fixation test, *ELISA* enzyme-linked immunoassay, *IF* immunofluorescence, *IFAT* indirect fluorescent antibody test, *LN* lymph node, *MAT* modified agglutination test, *PCR* polymerase chain reaction, *WAT* Wright’s agglutination test, *WB* western blot

### Crimean-Congo hemorrhagic fever

Crimean-Congo hemorrhagic fever is a disease caused by a Crimean-Congo hemorrhagic fever virus (CCHFV), a tick-transmitted orthonairovirus. It is a zoonotic disease that can cause potentially lethal systemic hemorrhagic disease in humans, with a mortality rate ranging between 3 and 30% of the humans infected (Ergonul 2012; Bente et al. 2013). Domestic and wild animals can be subclinically infected but are infectious for ticks feeding from them (Spengler et al. 2016). Autochthonous human clinical cases with associated mortality have been recently reported in the Iberian Peninsula (Negredo et al. 2017, 2021a, b), although it is yet unclear whether the virus was already present or its distribution area has lately spread (Spengler and Bente 2017). Ticks belonging to the genus *Hyalomma* spp. were traditionally considered the main vector of CCHFV (Spengler et al. 2016), but different CCHFV strains have been recently identified in Spain in multiple tick species of different genera obtained from the environment and domestic and wild animals (Moraga-Fernández et al. 2021; Sánchez-Seco et al. 2022), widening the epidemiological scenario of CCHFV transmission. Seroprevalences of antibodies against CCHFV reaching up to 100% have been detected in Iberian ibex Mediterranean populations, suggesting that CCHFV is endemic in the region at least since 2010, with a clustered distribution associated with high densities of bovine livestock (Espunyes et al. 2021; Carrera-Faja et al. 2022) (Table 3). Although CCHFV has not been detected in Iberian ibex nor in ticks carried by this species, the high seroprevalences reported warrant further research on the potential role of this species as a CCHFV reservoir and/or mechanical spreader of CCHFV-infected ticks, to assess the potential zoonotic risk.

### Abortive agents

A number of abortive agents can cause congenital perinatal mortality in wild ruminants. In Iberian ibex, the presence and/or contact with the abortive agents *Chlamydophila* spp., *Coxiella burnetii* (etiological agent of Q fever), *Leptospira pomona*, *Mycoplasma agalactiae*, *M. arginine* and *M. mycoides* subspecies *mycoides* (etiological agents of contagious agalactia), *Neospora caninum*, pestivirus (etiological agent of border disease), *Salmonella abortusovis*, Schmallenberg virus, and *Toxoplasma gondii* has been investigated and/or reported (Almería et al. 2007; González-Candela et al. 2007; León-Vizcaíno et al. 2008d; García-Bocanegra et al. 2012b; Astorga-Márquez et al. 2014; Junta de Andalucía 2015; Cano-Manuel 2017; Ruiz-Rodríguez 2018; Varela-Castro et al. 2018; Calero-Bernal et al. 2020; Gómez-Guillamón et al. 2020; Almería et al. 2021; González-Insa 2021; Jiménez-Ruiz et al. 2021) (Table 3). Although undoubtedly all these agents can affect population dynamics by decreasing fertility, birth rate, and kid survival, the demographic impact of these pathogens has not been studied nor reported in Iberian ibex populations. Moreover, if Iberian ibex populations are capable of maintaining the circulation of these pathogens as a reservoir, this may also suppose a risk and concern for sympatric domestic livestock.

The clinical outcomes and prevalences reported vary from failure to detect and/or identify the agent to high prevalences, so no consistent trend can be identified for abortive pathogens in Iberian ibex (Table 3). The prevalences reported probably depend on the particular agent and population studied; the objective of the diagnosis (contact through antibodies or identification of the etiological agent), the specific diagnostic methodology used, the specific ecosystem and environment inhabited by each ibex population studied, and the contact with and pressure by domestic livestock, among other factors. It is therefore difficult to establish a role as a reservoir or spillover host for Iberian ibex with regard to these pathogens, as well as the aforementioned effect on population demography and dynamics. However, since these diseases are shared with domestic livestock and some of them have zoonotic potential, increasing health surveillance at the interface between domestic and wild animals, including Iberian ibex, is warranted, as well as performing further specific studies aimed at elucidating the demographic effect of these infections in Iberian ibex population dynamics and the potential risk for domestic livestock.

### Bacterial and viral pneumoniae

Besides abortive agents, pneumoniae play a relevant and long-known role in population dynamics of wild mountain ungulates, either through yearly mortality of young animals during their first winter or through mortality outbreaks in adult individuals, usually linked to the exposition to new pathogens carried by domestic animals and/or changes in environmental or climatological conditions (Foreyt and Jessup 1982; Black et al. 1988; Gonzalez and Crampe 2001; Rudolph et al. 2007; Besser et al. 2008; Ytrehus et al. 2008; Posautz et al. 2014; Kock et al. 2018). There is an extended number of potential pathogens identified in pneumonia outbreaks in wildlife, including among other bacteria such as *Bibersteinia trehalosi* (formerly known as *Pasteurella trehalosi*), *Corynebacterium pyogenes* (most likely opportunistic), *Mannheimia glucosida*, *Mannheimia haemolytica*, *Moraxella bovis*, *Mycoplasma ovipneumoniae*, *Pasteurella multocida*, other hemolytic *Pasteurellaceae* or *Mannheimia* spp. or viruses as parainfluenza-3 virus (pneumovirus), and syncytial respiratory virus (respirovirus) (Foreyt and Jessup 1982; Black et al. 1988; Lavín et al. 2000b; Rudolph et al. 2007; Besser et al. 2008; Ytrehus et al. 2008; Dassanayake et al. 2010, 2013; Posautz et al. 2014; Kock et al. 2018). However, despite the long time elapsed since the causal relationship among pathogens, disease, mortality, and demographic effects has been established, there is still controversy about the relationship among the different pathogens identified and what combinations or action sequences cause disease and mortality and which do not (Dassanayake et al. 2010, 2013). Although the impact of pneumoniae on the population dynamics of other mountain ungulate species mainly by affecting recruitment is well known, this disease has not been thoroughly investigated in Iberian ibex, whose populations occupy different habitats and environments ranging from sea level to more than 3400 m above sea level (Pérez et al. 2002) and can therefore be challenged by pathogens causing pneumonia in different environmental conditions and seasons. A serological study was carried out in Iberian ibexes from southern Spain, revealing 8.1% and 1.3% seroprevalence against *P. multocida* A and *P. multocida* D, respectively (Cubero et al. 2002). Since pneumonia can cause mass mortalities leading to wiping out completely the affected populations and even leading almost to the extinction of common and abundant species (Ytrehus et al. 2008; Kock et al. 2018), further research on the etiology, pathogenesis, and demographic effects of these respiratory processes in wild mountain ungulates and particularly in Iberian ibex is required, in order to be able to prevent future disease and mortality outbreaks and improve their management if they occur, as well as to take into account their demographic effect for population management. In captive populations, stress-related immunosuppression, overcrowding and deficient ventilation, and changes in diet favor pneumonic and septicaemic outbreaks by *M. haemolytica*, *P. multocida* and *B. trehalosi*, and paramyxovirus (Espinosa et al. 2017c).

### Other infectious bacterial and viral diseases

Other pathogens have been punctually described in Iberian ibex without associated widespread distribution in the investigated population, disease outbreak, mortality, demographic effect, and/or potential risk for domestic livestock or humans (Antón et al. 1999, 2002; González-Candela et al. 2006; Revilla Calavia 2012; Calero-Bernal et al. 2020). Thus, low prevalence (1.25%) associated with domestic livestock has been described against *Escherichia coli* O157:H7, a potentially zoonotic bacterium (Navarro-González et al. 2015). *Staphylococcus* spp. has been cultivated from Iberian ibex, even with a 100% prevalence (González-Candela et al. 2006; Calero-Bernal et al. 2020), although no clear connection with pathology and population impact could be established. A new bacterium, *Streptococcus caprae* spp. nov., with phenotypic, biochemical, and phylogenetic differences with other *Streptococcus* species has been described in Iberian ibex (Vela et al. 2016). Antibodies against the Maedi-Visna virus were detected in a serosurvey in two out of 38 Iberian ibexes analyzed, accounting for a 5.3% prevalence (González-Insa 2021).

### Other non-lethal parasites

Apart from the parasites described above, widespread in Iberian ibex populations and/or related to disease and mortality outbreaks (*S. scabiei*, *O. caucasicus*, *B. ovis*, and *H. contortus*, see “Sarcoptic mange” and “Parasitic diseases”), more than 60 parasite species have been described in the Iberian ibex, including ectoparasites, gastrointestinal and pulmonary nematodes (with prevalences over 80%), coccidia, cestodes, trematodes, and protozoans (Rossi et al. 1994; Cano et al. 1996; Castellà et al. 1996; Pérez et al. 1996a, 2003, 2006; Antón et al. 1999, 2002; Granados et al. 2001; Refoyo et al. 2016; Calero-Bernal et al. 2020; Carrau et al. 2021a, b) (Table 4). More recently, intense tick infestations have been reported, which could increase in the future if global change provides conditions favoring their life cycle. Such intense tick burdens can affect the health and body condition of the host both per se through blood loss and by acting as vectors and/or amplifiers of other pathogens (Varela-Castro et al. 2018). The parasitic fauna of Iberian ibex forms a complex ecosystem where the different parasite species interact among them, with the host, and the environment, maintaining a dynamic balance that can lead to disease in the individual or even cause population epizootics as a result of disruptions in the system (Pérez et al. 2006).

**Table 4:** Parasites reported in Iberian ibex

Parasite	Prevalence	95% central CI (%)	Reference
**Abomasum**			
*Haemonchus contortus*	50.0% (7/14)	23.8 – 76.2	Rossi et al. (1994)
	14.3% (2/14)	0.0 – 32.6	Carrau et al. (2021a)
*Marshallagia dentispicularis*	28.6% (4/14)	4.9 – 52.2	Carrau et al. (2021a)
*Marshallagia marshalli*	35.7% (5/14)	10.6 – 60.8	Rossi et al. (1994)
	86.7% (13/15)	69.5 – 100.0	Pérez et al. (1996a)
	85.5% (94/110)	78.9 – 92.0	Antón et al. (2002), Pérez et al. (2006)
	78.5% (62/79)	69.4 – 87.5	Pérez et al. (2003)
	35.7% (5/14)	10.6 – 60.8	Carrau et al. (2021a)
*Marshallagia occidentalis*	14.3% (2/14)	0.0 – 32.6	Rossi et al. (1994)
	7.3% (8/110)	2.4 – 12.1	Antón et al. (2002), Pérez et al. (2006)
	22.8% (18/79)	13.5 – 32.0	Pérez et al. (2003)
*Nematodirus abnormalis*	0.9% (1/110)	0.0 – 2.7	Antón et al. (2002), Pérez et al. (2006)
	3.8% (3/79)	0.0 – 8.0	Pérez et al. (2003)
*Nematodirus davtiani*	20.0% (3/15)	0.0 – 40.2	Pérez et al. (1996a)
	21.8% (24/110)	14.1 – 29.5	Antón et al. (2002), Pérez et al. (2006)
	21.5% (17/79)	12.5 – 30.6	Pérez et al. (2003)
*Nematodirus oiratianus*	35.5% (39/110)	26.5 – 44.4	Antón et al. (2002), Pérez et al. (2006)
	2.5% (2/79)	0.0 – 6.0	Pérez et al. (2003)
*Ostertagia circumcincta*	100.0% (14/14)	100.0 – 100.0	Rossi et al. (1994)
*Ostertagia leptospicularis*	14.3% (2/14)	0.0 – 32.6	Carrau et al. (2021a)
*Ostertagia lyrate*	0.9% (1/110)	0.0 – 2.7	Antón et al. (2002), Pérez et al. (2006)
	1.3% (1/79)	0.0 – 3.7	Pérez et al. (2003)
*Ostertagia occidentalis*	7.1% (1/14)	0.0 – 20.6	Carrau et al. (2021a)
*0stertagia ostertagi*	80.0% (12/15)	59.8 – 100.0	Pérez et al. (1996a)
	14.5% (16/110)	8.0 – 21.1	Antón et al. (2002), Pérez et al. (2006)
	20.3% (16/79)	11.4 – 29.1	Pérez et al. (2003)
	0.0% (0/14)	0.0 – 0.0	Carrau et al. (2021a)
*Ostertagia trifurcata/pinnata*	64.3% (9/14)	39.2 – 89.4	Rossi et al. (1994)
*Spiculopteragia asymmetrica*	42.9% (6/14)	16.9 – 68.8	Carrau et al. (2021a)
*Spiculopteragia quadrispiculata*	21.4% (3/14)	0.0 – 42.9	Carrau et al. (2021a)
*Teladorsagia circumcincta*	85.5% (94/110)	78.9 – 92.0	Antón et al. (2002), Pérez et al. (2006)
	97.5% (77/79)	94.0 – 100.0	Pérez et al. (2003)
	92.9% (13/14)	79.4 – 100.0	Carrau et al. (2021a)
*Teladorsagia davtiani*	0.9% (1/110)	0.0 – 2.7	Antón et al. (2002), Pérez et al. (2006)
	1.3% (1/79)	0.0 – 3.7	Pérez et al. (2003)
	0.0% (0/14)		Carrau et al. (2021a)
*Teladorsagia trifurcata*	14.5% (16/110)	8.0 – 21.1	Antón et al. (2002), Pérez et al. (2006)
	46.8% (37/79)	35.8 – 57.8	Pérez et al. (2003)
	71.4% (10/14)	47.8 – 95.1	Carrau et al. (2021a)
*Trichostrongylus axei*	35.7% (5/14)	10.6 – 60.8	Rossi et al. (1994)
	0.9% (1/110)	0.0 – 2.7	Antón et al. (2002), Pérez et al. (2006)
	2.5% (2/79)	0.0 – 6.0	Pérez et al. (2003)
	35.7% (5/14)	10.6 – 60.8	Carrau et al. (2021a)
*Trichostrongylus vitrinus*	1.8% (2/110)	0.0 – 4.3	Antón et al. (2002), Pérez et al. (2006)
	2.5% (2/79)	0.0 – 6.0	Pérez et al. (2003)
**Small intestine**			
*Avitellina centripunctatta*	0.2% (1/489)	0.0 – 0.6	Antón et al. (2002), Pérez et al. (2006)
*Marshallagia marshalli*	20.9% (23/110)	13.3 – 28.5	Antón et al. (2002), Pérez et al. (2006)
	14.4% (12/83)	6.9 – 22.0	Pérez et al. (2003)
*Marshallagia occidentalis*	7.3% (8/110)	2.4 – 12.1	Antón et al. (2002), Pérez et al. (2006)
	3.6% (3/83)	0.0 – 7.6	Pérez et al. (2003)
*Nematodirus abnormalis*	28.6% (4/14)	4.9 – 52.2	Rossi et al. (1994)
	100.0% (15/15)	100.0 – 100.0	Pérez et al. (1996a)
	54.5% (60/110)	45.2 – 63.9	Antón et al. (2002), Pérez et al. (2006)
	42.2% (35/83)	7.9 – 76.4	Pérez et al. (2003)
	82.4% (14/17)	64.2 – 100.0	Carrau et al. (2021a)
*Nematodirus davtiani*	42.9% (6/14)	16.9 – 68.8	Rossi et al. (1994)
	57.3% (63/110)	48.0 – 66.5	Antón et al. (2002), Pérez et al. (2006)
	44.6% (37/83)	33.9 – 55.3	Pérez et al. (2003)
*Nematodirus filicollis*	100.0% (15/15)	100.0 – 100.0	Pérez et al. (1996a)
	1.8% (2/110)	0.0 – 4.3	Antón et al. (2002), Pérez et al. (2006)
	1.2% (1/83)	0.0 – 3.6	Pérez et al. (2003)
	11.8% (2/17)	0.0 – 27.1	Carrau et al. (2021a)
*Nematodirus oiratianus*	56.4% (62/110)	47.1 – 65.6	Antón et al. (2002), Pérez et al. (2006)
	47.0% (39/83)	36.3 – 57.7	Pérez et al. (2003)
*Nematodirus spathiger*	7.1% (1/14)	0.0 – 20.6	Rossi et al. (1994)
	2.7% (3/110)	0.0 – 5.8	Antón et al. (2002), Pérez et al. (2006)
	2.4% (2/83)	0.0 – 5.7	Pérez et al. (2003)
	47.1% (8/17)	23.3 – 70.8	Carrau et al. (2021a)
*Ostertagia ostertagi*	4.5% (5/110)	0.7 – 8.4	Antón et al. (2002), Pérez et al. (2006)
	3.6% (3/83)	0.0 – 7.6	Pérez et al. (2003)
*Teladorsagia circumcincta*	47.3% (52/110)	37.9 – 56.6	Antón et al. (2002), Pérez et al. (2006)
	31.3% (26/83)	21.3 – 41.3	Pérez et al. (2003)
*Teladorsagia trifurcata*	9.1% (10/110)	3.7 – 14.5	Antón et al. (2002), Pérez et al. (2006)
	7.2% (6/83)	1.7 – 12.8	Pérez et al. (2003)
*Trichostrongylus capricola*	85.7% (12/14)	67.4 – 100.0	Rossi et al. (1994)
	2.7% (3/110)	0.0 – 5.8	Antón et al. (2002), Pérez et al. (2006)
	1.2% (1/83)	0.0 – 3.6	Pérez et al. (2003)
	70.6% (12/17)	48.9 – 92.2	Carrau et al. (2021a)
*Trichostrongylus colubriformis*	50.0% (7/14)	23.8 – 76.2	Rossi et al. (1994)
	21.6% (8/37)	8.4 – 34.9	Pérez et al. (1996a)
	0.0% (0/17)	0.0 – 0.0	Carrau et al. (2021a)
*Trichostrongylus vitrinus*	21.4% (3/14)	0.0 – 42.9	Rossi et al. (1994)
	4.5% (5/110)	0.7 – 8.4	Antón et al. (2002), Pérez et al. (2006)
	3.6% (3/83)	0.0 – 7.6	Pérez et al. (2003)
	70.6% (12/17)	48.9 – 92.2	Carrau et al. (2021a)
**Large intestine**			
*Chabertia ovina*	52.9% (9/17)	29.2 – 76.7	Carrau et al. (2021a)
*Oesophagostomum venulosum*	36.3% (4/11)	7.9 – 64.8	Rossi et al. (1994)
	70.6% (12/17)	48.9 – 92.2	Carrau et al. (2021a)
*Skrjabinema ovis*	29.4% (5/17)	7.8 – 51.1	Carrau et al. (2021a)
*Skrjabinema* sp.	27.3% (3/11)	1.0 – 53.6	Rossi et al. (1994)
*Trichuris ovis*	9.1% (1/11)	0.0 – 26.1	Rossi et al. (1994)
	5.9% (1/17)	0.0 – 17.1	Carrau et al. (2021a)
**Faeces**			
*Eimeria aspheronica*	0.5% (2/379)	0.0 – 1.3	Antón et al. (2002), Pérez et al. (2006)
*Eimeria arloingi*	73.9% (280/379)	69.5 – 78.3	Antón et al. (2002), Pérez et al. (2006)
*Eimeria caprina*	5% (19/379)	2.8 – 7.2	Antón et al. (2002), Pérez et al. (2006)
*Eimeria capraovina*	5% (19/379)	2.8 – 7.2	Antón et al. (2002), Pérez et al. (2006)
*Eimeria christenseni*	10.0% (38/379)	7.0 – 13.1	Antón et al. (2002), Pérez et al. (2006)
	86.4% (19/22)	72.0 – 100.0	Pérez et al. (1996a)
*Eimeria folchijevi*	2.9% (11/379)	1.2 – 4.6	Antón et al. (2002), Pérez et al. (2006)
*Eimeria hirci*	1.1% (4/379)	0.0 – 2.1	Antón et al. (2002), Pérez et al. (2006)
*Eimeria ninakohlykimovae*	59.0% (13/22)	38.5 – 79.6	Pérez et al. (1996a)
	6.1% (23/379)	3.7 – 8.5	Antón et al. (2002), Pérez et al. (2006)
*Eimeria parva*	63.6% (14/22)	43.5 – 83.7	Pérez et al. (1996a)
*Eimeria* sp.	0.5% (2/379)	0.0 – 1.3	Antón et al. (2002), Pérez et al. (2006)
	97.7% (127/130)	95.1 – 100.0	Calero-Bernal et al. (2020)
*Moniezia expansa*	5.4% (2/37)	0.0 – 12.7	Pérez et al. (1996a)
	8.0% (39/489)	5.6 – 10.4	Antón et al. (2002), Pérez et al. (2006)
*Moniezia benedeni*	7.8% (38/489)	5.4 – 10.1	Antón et al. (2002), Pérez et al. (2006)
*Moniezia* sp.	10.0% (13/130)	4.8 – 15.2	Calero-Bernal et al. (2020)
*Bunostomum* sp.	2.5% (1/40)	0.0 – 7.3	Refoyo et al. (2016)
*Cooperia* sp.	2.5% (1/40)	0.0 – 7.3	Refoyo et al. (2016)
*Cystocaulus ocreatus*	16.3%*		Cano et al. (1996)
*Dictyocaulus filaria*	37.5%*		Cano et al. (1996)
	1.5% (2/130)	0.0 – 3.7	Calero-Bernal et al. (2020)
*Dictyocaulus* sp.	7.5% (3/40)	0.0 – 15.7	Refoyo et al. (2016)
*Muellerius capillaris*	66.0%*		Cano et al. (1996)
	97.3% (110/113)	94.4 – 100.0	Calero-Bernal et al. (2020)
*Muellerius* sp.	62.5% (25/40)	47.5 – 77.5	Refoyo et al. (2016)
*Nematodirus/Marshallagia* sp.	22.5% (9/40)	9.6 – 35.4	Refoyo et al. (2016)
*Nematodirus* sp.	50.0% (65/130)	41.4 – 58.6	Calero-Bernal et al. (2020)
*Ostertagia* sp.	7.5% (3/40)	0.0 – 15.7	Refoyo et al. (2016)
*Protostrongylus* sp.	4.5% (1/22)	0.0 – 13.2	Pérez et al. (1996a)
Protostrongylus rufescens*	21.4%		Cano et al. (1996)
*Skrjabinema* sp.	42.5% (17/40)	27.2 – 57.8	Refoyo et al. (2016)
*Strongyloides* sp.	17.5% (7/40)	5.7 – 29.3	Refoyo et al. (2016)
*Strongylidae*	93.1% (121/130)	88.7 – 97.4	Calero-Bernal et al. (2020)
*Teladorsagia* sp.	10.0% (4/40)	0.7 – 19.3	Refoyo et al. (2016)
*Toxocara vitulorum*	0.8% (1/130)	0.0 – 2.3	Calero-Bernal et al. (2020)
*Trichuris* sp.	2.4% (9/379)	0.8 – 3.9	Antón et al. (2002), Pérez et al. (2006)
	2.5% (1/40)	0.0 – 7.3	Refoyo et al. (2016)
	0.8% (1/130)	0.0 – 2.3	Calero-Bernal et al. (2020)
*Dicrocoelium dendriticum*	0.5% (2/379)	0.0 – 1.3	Antón et al. (2002), Pérez et al. (2006)
	20.8% (30/144)	14.2 – 27.5	Calero-Bernal et al. (2020)
*Dicrocoelium* sp.	2.5% (1/40)	0.0 – 7.3	Refoyo et al. (2016)
*Fasciola hepatica*	1.3% (5/379)	0.2 – 2.5	Antón et al. (2002), Pérez et al. (2006)
*Paramphistomum* sp.	0.5% (2/379)	0.0 – 1.3	Antón et al. (2002), Pérez et al. (2006)
	9.1% (2/22)	0.0 – 21.1	Pérez et al. (1996a)
**Liver**			
*Fasciola hepatica*	6.7% (1/15)	0.0 – 19.3	Pérez et al. (1996a)
	1.7% (8/478)	0.5 – 2.8	Antón et al. (2002), Pérez et al. (2006)
**Lungs**			
*Cystocaulus ocreatus*	20.0% (3/15)	0.0 – 40.2	Pérez et al. (1996a)
	32.1% (267/831)	29.0 – 35.3	Antón et al. (2002), Pérez et al. (2006)
	26.3% (5/19)	6.5 – 46.1	Carrau et al. (2021b)
*Dictyocaulus filaria*	1.2% (10/831)	0.5 – 1.9	Antón et al. (2002), Pérez et al. (2006)
	0.0% (0/19)	0.0 – 0.0	Carrau et al. (2021b)
*Echinococcus granulosus*	0.2% (1/479)	0.0 – 0.6	Antón et al. (2002), Pérez et al. (2006)
*Muellerius capillaris*	26.7% (4/15)	4.3 – 49.0	Pérez et al. (1996a)
	74.2% (617/831)	71.3 – 77.2	Antón et al. (2002), Pérez et al. (2006)
	84.2% (16/19)	67.8 – 100.0	Carrau et al. (2021b)
*Neostrongylus linearis*	78.9% (15/19)	60.6 – 97.3	Carrau et al. (2021b)
*Neostrongylus* sp.	31.3% (260/831)	28.1 – 34.4	Antón et al. (2002), Pérez et al. (2006)
*Protostrongylus* sp.	35.3% (293/831)	32.0 – 38.5	Antón et al. (2002), Pérez et al. (2006)
	57.9% (11/19)	35.7 – 80.1	Carrau et al. (2021b)
**Diaphragm**			
*Sarcocystis* sp.	80% (24/30)	65.7 – 94.3	Pérez et al. (1996a)
	27.4% (118/430)	23.2 – 31.7	Antón et al. (2002), Pérez et al. (2006)
	84.0% (42/50)	73.8 – 94.2	Calero-Bernal et al. (2020)
**Peritoneum**			
*Avitellina* sp.	6.7% (1/15)	0.0 – 19.3	Pérez et al. (1996a)
*Taenia hydatigena*	27.1% (130/479)	23.2 – 31.1	Antón et al. (2002), Pérez et al. (2006)
	6.9% (10/144)	2.8 – 11.1	Calero-Bernal et al. (2020)
**Brain**			
*Multiceps multiceps*	1.1% (2/180)	0.0 – 2.6	Pérez et al. (1996a)
*Taenia multiceps*	0.3% (2/582)	0.0 – 0.8	Antón et al. (2002), Pérez et al. (2006)
**Skin**			
*Dermacentor marginatus*	**		Castellà et al. (1996)
	2.1% (10/475)	0.8 – 3.4	Antón et al. (2002), Pérez et al. (2006)
*Dermacentor reticulatus*	0.6% (3/475)	0.0 – 1.3	Antón et al. (2002), Pérez et al. (2006)
*Haemaphysalis sulcata*			Castellà et al. (1996)
	32.2% (153/475)	28.0 – 36.4	Antón et al. (2002), Pérez et al. (2006)
	10.9% (15/138)	5.7 – 16.1	Calero-Bernal et al. (2020)
*Haemaphysalis punctata*	**		Castellà et al. (1996)
*Hyalomma beneditai*	18.9% (36/190)	13.4 – 24.5	Pérez et al. (1996a)
*Hyalomma marginatum marginatum*	5.1% (7/138)	1.4 – 8.7	Calero-Bernal et al. (2020)
*Ixodes ricinus*	20.0% (38/190)	14.3 – 25.7	Pérez et al. (1996a)
	4.2% (20/475)	2.4 – 6.0	Antón et al. (2002), Pérez et al. (2006)
	10.9% (15/138)	5.7 – 16.1	Calero-Bernal et al. (2020)
*Rhipicephalus bursa*	**		Castellà et al. (1996)
	42.7% (203/475)	38.3 – 47.2	Antón et al. (2002), Pérez et al. (2006)
	52.2% (72/138)	43.8 – 60.5	Calero-Bernal et al. (2020)
*Rhipicephalus pusilus*	15.8% (30/190)	10.6 – 21.0	Pérez et al. (1996a)
*Bovicola crassipes*	14.2% (27/190)	9.2 – 19.2	Pérez et al. (1996a)
	20.2% (96/475)	16.6 – 23.8	Antón et al. (2002), Pérez et al. (2006)
*Linnognathus* sp.	1.1% (2/190)	0.0 – 2.5	Pérez et al. (1996a)
*Linognathus stenopsis*	1.1% (5/475)	0.1 – 2.0	Antón et al. (2002), Pérez et al. (2006)
*Psoroptes* sp.	0.6% (3/475)	0.0 – 1.3	Antón et al. (2002), Pérez et al. (2006)
*Trombicula* sp.	1.6% (3/190)	0.0 – 3.4	Pérez et al. (1996a)
	0.4% (2/475)	0.0 – 1.0	Antón et al. (2002), Pérez et al. (2006)
*Straelensia cynotis*	One case report		Cevidanes et al. (2019)
**Blood**			
*Anaplasma ovis*	4.5% (5/110)	0.7 – 8.4	Antón et al. (2002), Pérez et al. (2006)
*Eperytrozoon ovis*	1.8% (2/110)	0.0 – 4.3	Antón et al. (2002), Pérez et al. (2006)
*Ehrlichia phagocytophila*	0.9% (1/110)	0.0 – 2.7	Antón et al. (2002), Pérez et al. (2006)
*Theileria ovis*	1.8% (2/110)	0.0 – 4.3	Antón et al. (2002), Pérez et al. (2006)

^*^ Cano et al. (1996) reported prevalence but did not provide accurate sample size information to allow for estimation of confidence intervals

^**^ Castellà et al. (1996) reported presence but did not provide prevalence values

### Non-infectious diseases

Dental and skull abnormalities have been reported in Iberian ibex, both in current populations and in paleontological remnants (Vigal and Machodorm 1987; Gómez-Olivencia et al. 2011). Although anecdotal and with little impact on the population dynamics of the species, tumors have been reported in Spanish ibex. Thyroid carcinoma, cutaneous horn, pheochromocytoma, intestinal leiomyoma, and ruminal papilloma have been described in Iberian ibexes after necropsy during regular wildlife health surveillance (Arnal et al. 2006). Two gastrointestinal tumors were found in two free-ranging Iberian ibexes apparently healthy otherwise (Velarde et al. 2008), and disseminated melanoma concurrent with pheochromocytoma severely affected an old male to euthanasia (Arnal and Fernández de Luco 2017).

## Diseases related to or favored by captivity

Captive Iberian ibex populations are kept in enclosures or at high density in fenced estates either for conservation purposes or for exploitation as game resource (Granados et al. 1996b; Espinosa et al. 2017c). The higher host densities associated with these conditions can favor pathogen circulation, generating a more intense infection pressure that can explain the higher prevalence and severity and even the different epidemiological nature of disease outbreaks in captive Iberian ibex populations as compared to free-ranging populations, as described for caseous lymphadenitis in the “Caseous lymphadenitis” section and for pneumonia in the “Bacterial and viral pneumoniae” section (Colom-Cadena et al. 2014; Espinosa et al. 2017c; Varela-Castro et al. 2017). Moreover, other diseases mentioned below have not been described or do not cause symptomatology in free-ranging Iberian ibex populations but have been detected and caused disease only in captive populations (Espinosa et al. 2017c).

Enterotoxemia or “pulpy kidney disease” is a disease caused by the epsilon toxins of the gram-positive bacterium *Clostridium perfringens* type D. The disease is normally related to qualitative and/or quantitative changes in diet leading to intestinal dysbiosis, favoring bacterium encapsulation and toxin production (Uzal and Kelly 1996; García et al. 2013; Espinosa et al. 2017c). Although such diet changes can also happen seasonally or because of habitat modifications in free-ranging Iberian ibex populations, this is more frequent and sudden in captive populations where diet is managed by humans, increasing the probability and severity of clostridiosis (Espinosa et al. 2017c). Quarantine and vaccination (at the entry to captivity and annually), a fiber-rich diet without sudden changes, and avoiding stress are the recommended prophylactic measures. If disease occurs in a captive Iberian ibex population, treatment should include antibiotics and the aforementioned fiber-rich diet (Uzal and Kelly 1996; García et al. 2013; Espinosa et al. 2017c).

**Fig. 7:**
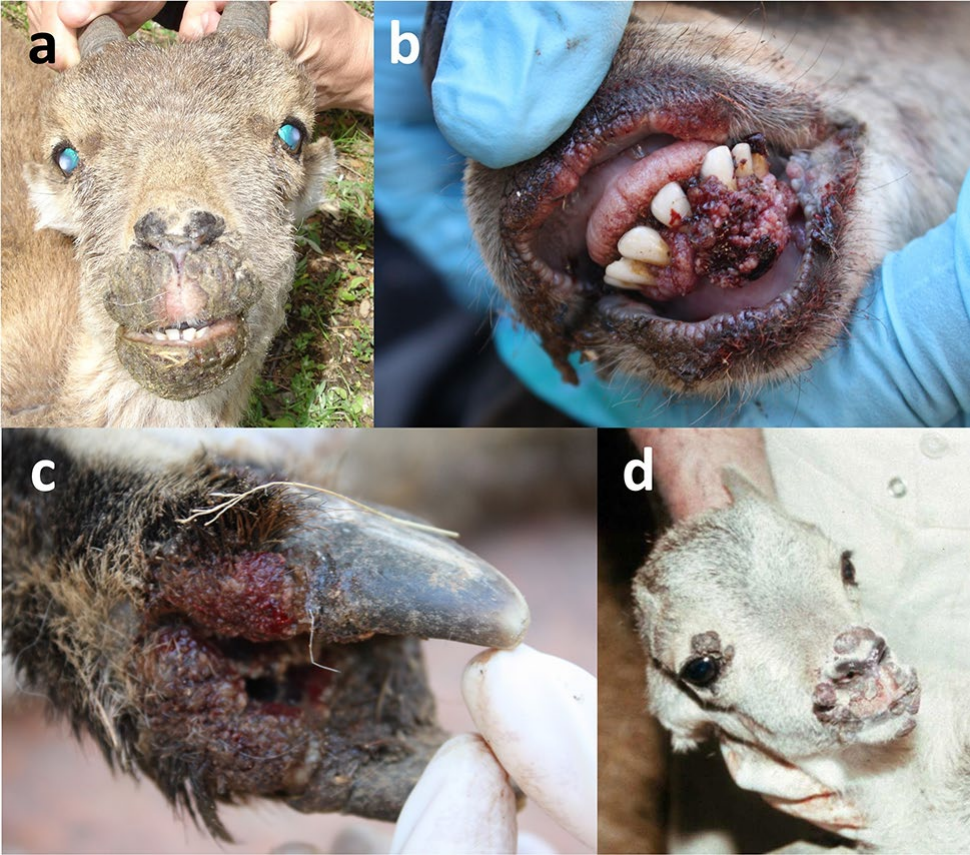
Adult Iberian ibex female (**a**–**c**) and kid (**d**) with crusted skin lesions affecting the commissures of the mouth, peripheral lip epidermis (**a**, **d**), oral mucosa (**b**), and interdigital area (**c**), consistent with severe proliferative, pustular, and ulcerative dermatitis compatible with orf virus infection (contagious ecthyma)

Contagious ecthyma is a disease caused by the orf virus, included in the genus Parapoxvirus. This disease affects both domestic and wild small ruminants and is characterized by proliferative dermatitis with papules and pustules, usually affecting the mouth, lips, and nose, although the lesions can extend to the oral cavity, esophagus, abomasum, skin of the face, hooves, flanks, scrotum, perianal region, preputium, vulva, and mamma (Fig. 7). The clinical signs are more severe in kids, and the infection can complicate with secondary bacterial infections leading to death, although it is normally self-limiting. It is considered a professional zoonosis, although the clinical course is also benign and self-limiting in humans (Spyrou and Valiakos 2015). The stress associated with captivity seems to increase the morbidity and the clinical severity of the disease (Junta de Andalucía 2015). However, antibodies against this virus were detected in the sera of 11 out of 450 (2.4%) Iberian ibexes investigated in southern Spain (Cubero et al. 2002), indicating that the virus probably circulates asymptomatically or subclinically in free-ranging populations. Occasional clinical cases (Camacho et al. 2017) and a disease outbreak affecting both adults and kids with at least one case of associated mortality (Estruch et al. 2020) have been described in free-ranging Iberian ibex populations. Management of contagious ecthyma outbreaks in captive populations includes isolation and treatment with antiseptics and antibiotics of the affected individuals, while prophylaxis is based on facility and equipment disinfection, since there is not an effective prophylactic vaccine (Spyrou and Valiakos 2015; Espinosa et al. 2017c).

Footrot or infectious pododermatitis is a disease affecting the hooves of domestic and wild ungulates, with the involvement of bacteria such as *Dichelobacter* (*Bacteroides*) *nodosus*, *Fusobacterium necrophorum*, *Arcanobacterium* (*Trueperella*) *pyogenes*, and *Treponema* spp. (Lavín et al. 2004; Frosth et al. 2015; Espinosa et al. 2017c; Rzewuska et al. 2019). Although footrot has not been reported in free-living Iberian ibex, in captive populations, this disease is favored by the increase in concentration and the higher survival of these bacteria in moist soils with abundant organic contents (Muzafar et al. 2015), as well as by hoof wounds related to overgrowth because of lower wear and softening due to humid conditions (Espinosa et al. 2017c). Treatment of outbreaks in captive Iberian ibex populations should include hoof trimming with the removal of dead tissue, systemic antibiotics, local footbaths with disinfectants, and soil management to prevent moisture and accumulation of organic matter. Prophylactic vaccination can be considered if prevalence is high and/or outbreaks are recurrent (Frosth et al. 2015; Muzafar et al. 2015; Espinosa et al. 2017c).

Finally, *A.* (*T.*) *pyogenes* and/or *Staphylococcus aureus* subsp. *anaerobius* can cause suppurative lesions. These bacteria are normally present in the skin and mucosae and can enter the body through wounds, producing multiple abscesses in the inner organs such as the lymph nodes, lungs, liver, kidneys, and/or central nervous system, among others, which can cause death (Espinosa et al. 2017c; Rzewuska et al. 2019). Although this is a sporadic disease in free-ranging populations, captivity conditions can increase skin trauma due to wounds caused by the facility, increased intraspecific fights, or increased moisture, favoring the casuistic for pyogenic infection (Espinosa et al. 2017c). Quarantine isolation with surgical debriding and antibiotic treatment can be effective in initial stages, but in advanced cases, prognosis is poor. Facility design and careful handling to avoid injuries combined with early healing and disinfection of wounds are likely the best prophylactic options (Espinosa et al. 2017c).

## Future research on Iberian ibex diseases

The comparison of the existent knowledge on sarcoptic mange and other diseases in Iberian ibex identifies the gaps to complete through future research. The knowledge on pathogenesis, host immune response, and pathogen-host relationship for sarcoptic mange overcomes the research performed in these aspects for all the other diseases together. While the performance, usefulness on the field and with handled ibexes, specificity, and sensitivity of diagnostic methods have been assessed for sarcoptic mange (Arenas et al. 2002; Granados et al. 2011; Alasaad et al. 2012, 2015; Mounsey et al. 2012; Ráez-Bravo et al. 2016; Valldeperes et al. 2019; Pérez et al. 2021), such knowledge is limited if existing at all for the other diseases reported in Iberian ibex. Therefore, research on the pathogenesis, epidemiology, treatment, and management of diseases in Iberian ibex populations can be hampered by diagnostic limitations. Further research should be aimed at developing, testing, validating, improving, and innovating diagnostic methods, particularly non-invasive and telemetric methods which could be applied in free-ranging individuals and populations. Despite research on sarcoptic mange predominates the investigations of Iberian ibex diseases, the scientific and manager community is far from agreeing on the best management of the disease, so further research on the efficacy and applicability of the treatment and population management of sarcoptic mange in Iberian ibex should continue to be a priority. Such research should include prophylactic measures, not only for sarcoptic mange but also for the rest of Iberian ibex diseases. Finally, the study and detection of coinfection patterns, studying the interaction among different pathogens and the host, should be key to understand disease epidemiology in Iberian ibex, as it is for sympatric wild ungulates (Alasaad et al. 2008; González-Candela et al. 2009; García-Bocanegra et al. 2012b).

## Conclusions

This review compiles the current knowledge available on the diseases of Iberian ibex, which has allowed the detection of gaps in knowledge with regard to the diseases that may affect this species and their management. Overall, the information presented draws the major guidelines for the future of disease research in this species and the consequent management applications, including the diseases to monitor and the best management measures.

While sarcoptic mange is the main health threat to naïve Iberian ibex populations, associated with morbidity and mortality with demographic impact, most of the affected populations recover from the initial outbreaks and achieve an enzootic status, where the disease no longer hampers population viability. Thus, applying empiric measures to manage this disease without scientific validation may not only be useless or even counterproductive, but can also negatively affect the evolution from epidemic outbreak to enzootic status by eliminating potentially resistant individuals. Moreover, apart from being inefficient, massive on-field administration of antiparasitic drugs (namely, macrocyclic lactones) intended for individual parenteral use is a major animal welfare, environmental, and health concern. Since domestic livestock has been identified as the origin of sarcoptic mange outbreaks in Iberian ibex, surveillance and control of this disease in small ruminant livestock sympatric with Iberian ibex are probably more efficient management options.

The review of other diseases reported in Iberian ibex raises doubts about the rationale for the diseases included in the health surveillance of this species, such as tuberculosis or brucellosis, since Iberian ibex seems not to be a reservoir for these diseases and even rarely if ever affected by them. Instead, other diseases that might be relevant in the health surveillance of Iberian ibex populations deserve further research and monitoring to elucidate their potential effects in the population dynamics of the species.

## Data Availability

All the data used to generate this review are publicly available. The authors will share information on data location upon request.
